# Self-controlled designs in pharmacoepidemiology involving electronic healthcare databases: a systematic review

**DOI:** 10.1186/s12874-016-0278-0

**Published:** 2017-02-08

**Authors:** Nathalie Gault, Johann Castañeda-Sanabria, Yann De Rycke, Sylvie Guillo, Stéphanie Foulon, Florence Tubach

**Affiliations:** 1 APHP, Département d’Epidémiologie Biostatistiques et Recherche Clinique, Hôpital Bichat, 75018 Paris, France; 2 Université Paris Diderot, Sorbonne Paris Cité, UMR 1123 ECEVE, 75018 Paris, France; 3 INSERM CIC-EC 1425, Hôpital Bichat, 75018 Paris, France; 4 APHP, Département Biostatistiques Santé Publique et Information Médicale, Centre de Pharmaco-épidémiologie de l’AP-HP, Hôpital Pitié-Salpétrière, 75013 Paris, France; 5 Biostatistics unit, Gustave Roussy, 94800 Villejuif, France; 6 CESP, Université Paris-Sud, UVSQ, INSERM, Université Paris-Saclay, 94800 Villejuif, France; 7 Université Pierre et Marie Curie, Sorbonne Universités, 75013 Paris, France

**Keywords:** Pharmacoepidemiology, Self-controlled designs, Observational studies, Databases, Systematic review

## Abstract

**Background:**

Observational studies are widely used in pharmacoepidemiology. Several designs can be used, in particular self-controlled designs (case-crossover and self-controlled case series). These designs offer the advantage of controlling for time-invariant confounders, which may not be collected in electronic healthcare databases. They are particularly useful in pharmacoepidemiology involving healthcare database. To be valid, they require the presence of some characteristics (key validity assumptions), and in such situations, these designs should be preferred. We aimed at describing the appropriate use and reporting of the key validity assumptions in self-controlled design studies.

**Methods:**

Articles published between January 2011 and December 2014, and describing a self-controlled study design involving electronic healthcare databases were retrieved. The appropriate use (fulfilment of key assumptions) was studied in terms of major (abrupt onset event, rare or recurrent event, and intermittent exposure) and minor assumptions (those for which the design can be adapted).

**Results:**

Among the 107 articles describing a self-controlled design, 35/53 (66%) case-crossover studies, and 48/55 (87%) self-controlled case series fulfilled the major validity assumptions for use of the design; 4/35 and 14/48 respectively did not fulfill the minor assumptions. Overall, 31/53 (58%) case-crossover studies and 34/55 (62%) self-controlled case series fulfilled both major and minor assumptions. The reporting of the methodology or the results was appropriate, except for power calculation.

**Conclusions:**

Self-controlled designs were not appropriately used in34% and 13% of the articles we reviewed that described a case-crossover or a self-controlled case series design, respectively. We encourage better use of these designs in situations in which major validity assumptions are fulfilled (i.e., for which they are recommended), accounting for situations for which the design can be adapted.

**Electronic supplementary material:**

The online version of this article (doi:10.1186/s12874-016-0278-0) contains supplementary material, which is available to authorized users.

## Background

Pharmacoepidemiology aims at assessing the risk and benefit of pharmaceuticals in real-world populations [1]. Computerized medical databases are increasingly being used for real life post-marketing observational studies [2], and have several advantages: (i) the potential for studying a very large sample size, thereby allowing for study of rare events or exposures; (ii) the availability of data for older adults, children, patients with low resources, and nursing-home residents, who are most often under-represented in clinical trials [3]; (iii) the inclusion of off-label prescriptions; and (iv) data are prospectively collected [4]. However, data are usually collected for purposes other than research (administrative or healthcare management), so the databases frequently lack information on some potential confounding factors (e.g., genetics, body mass index, smoking status, alcohol consumption, or medical history and comorbidities) [2]. In this context, in which many confounders may not be collected, self-controlled designs are an interesting option in observational studies of pharmaceuticals [5]. Self-controlled designs are based only on cases, which then act as their own control (i.e., they consist in within-patient comparison between different periods of time). Their main advantage is that time-invariant confounders that act multiplicatively on the baseline rates are inherently controlled for. As Nordmann et al. reported in a systematic review [6], these designs mainly include the case-crossover design [7, 8] and the self-controlled case-series [9–11] and were developed to study the short term effect of transient-exposures and abrupt onset events. Indeed, because of the self-matched design, the risk estimation includes only data for patients who switch their exposure status over time (i.e., from exposed to unexposed, or vice versa). With sustained exposure, the opportunity for exposed patients to become unexposed is reduced, therefore leading to a smaller number of patients with “discordant” exposure status, and reduced power. Moreover, the study of sustained exposures over longer periods is subject to time-varying confounding that needs to be addressed. Studies of insidious-onset events (e.g., depression, cancer, autism) are subject to misclassification bias because of uncertainty in the onset date of the outcome. Self-controlled designs require that some other validity assumptions be fulfilled. For case-crossover design, the opportunity for exposure should be the same during the case and control time periods (e.g., for car crashes or the risk of alcohol consumption, the control period should be the same day of the week, because driving or drinking behaviour may vary from weekdays to weekends [8, 12, 13]), and there should not be any time trend in exposure. For self-controlled case series, two consecutive events should be independent if they are recurrent; the probability of further exposure should not be affected by a previous event; and the event should not affect the short-term mortality probability. Because of methodological developments, these designs have become applicable in more situations, by weakening the assumptions they require, such as the possibility to study time trend in exposure [14, 15], event-dependant exposure [16, 17], inter-dependant recurrences [18], or event-dependant observation periods [16, 19].

Several systematic reviews have examined the use and reporting of case-crossover designs only [20], self-controlled case series only [21] or self-controlled studies in general [6]. Recently, we showed that self-controlled designs are rarely used in pharmacoepidemiology [22]. We did not find any recent study focusing on the appropriate use of self-controlled designs in pharmacoepidemiology involving electronic healthcare databases, with regard to major and minor validity assumptions.

Here we aimed to assess the appropriate use of self-controlled designs, in terms of their validity assumptions in pharmacoepidemiology involving electronic healthcare databases and to update the Nordmann et al. review [6]. To achieve this goal, we performed a systematic review to describe whether the required characteristics for the use of the designs were fulfilled and adequately reported in published articles.

## Methods

### Search

For the systematic review, we searched MEDLINE via Pubmed for English and French articles of self-controlled studies (case-crossover or self-controlled case series) involving electronic healthcare databases that were published from January 1, 2011 to December 31, 2014. The search keywords are reported in Additional file 1: S1. Papers reporting the study of safety or efficacy of a medical product that used a self-controlled design and involving electronic healthcare databases were included. We excluded articles of studies not examining a drug or device, methodological articles (design development or study protocol), drug utilization or prescription studies (drug utilization or drug prescription patterns), descriptive or case-series studies (prevalence or incidence of disease), clinical practice evaluation (quality improvement in clinical practice), and articles not describing a self-controlled design or not involving electronic healthcare databases.

### Data collection

The data were collected by three readers who used a standardized extraction form (Additional file 1: S2) based on the STROBE recommendations [23] and on previous systematic reviews of designs in pharmacoepidemiology [6, 22].

The collected data focused on the characteristics of exposures and events and on the study designs. The *“exposure characteristics”* section contained information about the type, prevalence and characteristic of the exposure (*i.e.* one shot, such as vaccines; transient for a few days, such as antimicrobial therapy or analgesics; intermittent on a specified frequency, such as chemotherapy; or sustained, such as long term use of hypoglycaemic agents). In several cases, we reclassified the sustained exposures as transient, when the event of interest was explicitly hypothesised to occur shortly after the drug initiation (product initiation for incident user designs), or intermittent, when we considered that a high opportunity of switch from exposed to unexposed status (or vice versa) can be assumed over the observation period (especially when the risk period was equal and non-inferior^1^ to the prescription period). When no classification was possible after consensus, the exposure classification was considered unclear. The *“event characteristics”* section contained information about the type, definition, prevalence or incidence, and characteristics of the event, such as the potential for recurrence (unique, such as hip fracture, or recurrent, such as seizures), and its onset (abrupt, such as car crash, or insidious, such as depression). An event was considered recurrent if it was likely to occur several times within the same patient during the observation period (because recurrence was clearly reported in the article or such an event is usually considered recurrent, such as febrile convulsions). An event was considered rare if its prevalence in the source study population was less than 5%. Subjective items (*i.e.* type of exposure, event onset or recurrence) were discussed among the authors to reach consensus. The *“design”* section included information about the type (self-controlled case series or case-crossover) and characteristics of the study design: for case-crossover studies, definition of time windows; opportunity for exposure during the case and control periods, existence, reporting and consideration in the analysis of a time trend in exposure; and for self-controlled case series, independent recurrences in case of recurrent events, independence of exposure with respect to the event, and independence of the short-term mortality risk with respect to the event).

#### Major validity assumptions

For self-controlled designs (*i.e.* required characteristics that must be fulfilled for the self-controlled design to be valid) were based on the methodological studies for the corresponding designs [7–10]. For case-crossover studies, these characteristics are a transient or intermittent exposure, an abrupt-onset event, and a rare event. For self-controlled case series, they are a transient or intermittent exposure, an abrupt-onset event, and a rare and/or recurrent event.

#### Minor validity assumptions

For self-controlled designs (*i.e.* situations that can threaten the design validity but when the design can be adapted to be valid in light of recent methodological developments) were as follows: for case-crossover studies, the opportunity for exposure should be the same during the case and control time periods, and there should not be any time trend in exposure. We considered that a time trend existed if it was clearly reported by the authors or, when not clearly reported, if we considered that such trend could be suspected in the study setting (e.g., in the study of a drug shortly after market authorization (24) or the study of drugs during pregnancy especially when control periods are chosen before pregnancy because prescription patterns change after conception). For exposures with a time trend, we considered that the time trend was accounted for when the authors used a case-time control design or another appropriate design (such as bidirectional case-crossover design or case-case-time-control design) [14, 15, 24, 25]. For self-controlled case series, two consecutive events should be independent if they are recurrent; otherwise the design should be adapted as appropriate, as in considering only the first event [10, 18]. Also, the probability of further exposure should not be affected by a previous event (*i.e.* event-independent exposure assumption) or the design should be adapted as appropriate, as in excluding person-times before exposure [11, 16, 17]. Finally, the event should not affect the short-term mortality probability, or the analysis should be adapted by involving the time interval between the event and end of the actual observation period [11, 16, 19].

All assumptions were mainly assessed on what the authors reported in their paper. With no mention of these assumptions in the article, the assessment was based on our own judgement, after consensus: in particular, the appropriateness of these assumptions can be deduced for particular studied events (e.g., myocardial infarction, falls, or febrile convulsions) by referring to several methodological studies with applications to such events [7, 26, 27]. When we could establish no clear conclusion, the assumption was considered unclear.

Of note, we did not consider that the design adaptation proposed by Wang et al. to study sustained exposures or insidious-onset outcomes (i.e., prolonged exposure windows) [28] allows for consider them minor validity assumptions in all situations. In fact, there is still a validity threat when there is a small probability of switching between exposure statuses within the observation window or when the hypothesised effect is of a cumulative nature with a delayed onset. In the former setting, this design adaptation could lead to loss of statistical power (increasing type II error and false negatives) because of too many rare discordant cases in case-crossover studies or unexposed cases in self-controlled case series. Also, the probability for the occurrence of time-varying within-person confounders is high, so self-controlled designs not recommended as compared to cohort-based approaches in such situations.

For articles with several outcomes, we considered the assumptions valid if they were fulfilled for at least one outcome. Finally, we considered that a study did not fulfil minor assumptions if at least one of the assumptions was violated.

### Quality of reporting

We examined whether the recommendations from Nordmann *et al.* [6] were applied for quality of reporting. These recommendations include the reporting of the fulfilment of key assumptions (whether the setting was valid for the design implementation in terms of major and minor assumptions); the definition (number and duration) of the case period (for case-crossover) or risk period (for self-controlled case series) and the control periods; the appropriate statistical model (*i.e.* conditional logistic regression model or conditional Poisson regression model for case-crossover [29, 30], and conditional Poisson regression model, Cox’s stratified proportional hazards model or conditional logistic regression model for self-controlled case series [30]), an *a priori* sample size calculation (or power calculation, because sample size cannot be chosen in healthcare database research); the appropriate effect estimator (*i.e.* odds ratio for case-crossover and incidence rate ratio for self-controlled case series) with a measure of variability (confidence interval or standard deviation); unadjusted and adjusted estimators; the person-time in each risk and control period for self-controlled case series; and finally, the reporting of any sensitivity analyses.

### Statistics

The data are described as number (%). Concerning the exposure characteristics, “one shot”, “transient” and “intermittent” exposure categories were grouped as “intermittent”. To describe the appropriate of use of the self-controlled designs, we determined whether the major validity assumptions were fulfilled. Furthermore, we determined whether fulfilment of the minor validity assumptions was reported in articles reporting valid major assumptions and also overall. When the fulfilment of the assumptions was unclear, we considered them valid in our main analysis. We further performed a sensitivity analysis considering such unclear assumptions as invalid. Another sensitivity analysis was performed considering only one article in case of several papers from the same author(s) with similar objectives. The quality of reporting was also reported. Analyses involved use of R 2.15.2 (The R Foundation for Statistical Computing). The results of our systematic review were reported according to the PRISMA guidelines [31].

## Results

**Fig. 1:**
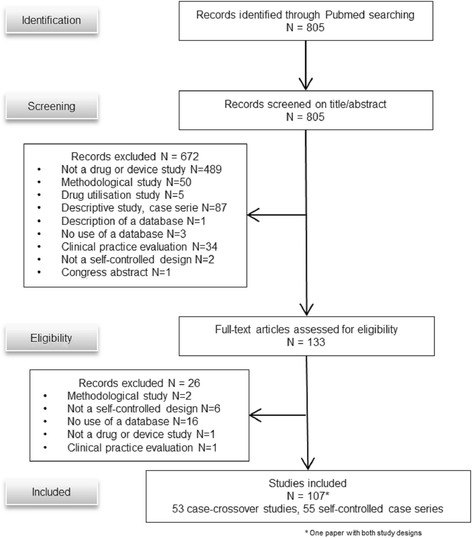
Flow chart of the selection of articles

We identified 107 articles describing self-controlled designs: 53 case-crossover and 55 self-controlled case series (one study used both designs, Fig. 1). The list of included articles is reported in Additional file 1: S3. Their characteristics are reported in Table 1. All papers were of drug safety.

**Table 1:** Characteristics of 107 studies with self-controlled designs

Publication journal topic categories	
Specialized journal	48 (45)
Pharmacology or epidemiology journal	43 (40)
General medicine journal	16 (15)
Studied exposure	
Vaccines	30 (28)
Psychotropic medications	25 (23)
Cardiovascular drugs (including anticoagulant or anti-platelet, antihypertensive and hypoglycaemic drugs)	15 (14)
Anti-infective agents	9 (8)
Analgesics	7 (7)
Gastroenterologic medications	6 (6)
Others	15 (14)
Studied event^a^	
Cardiovascular event	29 (27)
Neuro / psychological event	16 (15)
Hospital admission or emergency visit	13 (12)
Gastrointestinal event	12 (11)
Fractures, injury	8 (7)
Pneumological event	7 (7)
Traffic accident	6 (6)
Fall	4 (4)
Nephrological event	4 (4)
Deep vein thrombosis / pulmonary embolism	3 (3)
Other	10 (9)

Data are reported as n (%)

^a^Total can be more than 100% if several outcomes were studied in the same study

The fulfilment of the major and minor validity assumptions for the use of a self-controlled design is described in Table 2 and reported in details in Additional file 1: S4a and S4b. Overall, 18 (34%) articles using a case-crossover design and 7 (13%) articles using a self-controlled case series did not fulfil all of the major validity assumptions for their use: most frequently, the studies examined the effect of a sustained exposure (e.g., antihypertensive drugs or platelet aggregation inhibitors in cardiovascular primary prevention), and 3 examined an event with an insidious onset (e.g., depression, chronic fatigue syndrome or congenital malformation). Classification of exposure was unclear in 8 articles using a case-crossover design and 1 article using a self-controlled case series design. Among the 35 case-crossover studies with valid major assumptions, 4 did not fulfil the minor ones: a time trend in exposure was not accounted for in 3, and the opportunity of exposure could vary between case and control periods in another. Among the 48 self-controlled case series with valid major assumptions, 14 did not fulfil the minor ones: 1 study examined an event with recurrences that were not independent (i.e., migraine), 6 studies violated the assumption of event-independent exposure and 8 studies examined an outcome that could censor the observation period (such as myocardial infarction, stroke, heart failure). An exposure temporal trend was unclear or not reported in 22 articles using a case-crossover design. Overall, all the assumptions were fulfilled in 31 (58%) case-crossovers studies and 34 (62%) self-controlled case series. Considering unclear assumptions as invalid led to smaller proportions of appropriate studies, especially for case-crossover designs (Additional file 1: S5). Of note, a sensitivity analysis considering only one article when several from the same author(s) with similar objectives were included in the systematic review showed similar results (Additional file 1: S6).

**Table 2:** Fulfilment of the validity assumptions for the use of a self-controlled design

	Case-crossover	Self-controlled case-series
	*N* = 53	*N* = 55
Major assumptions:		
Type of studied exposure		
Intermittent/transient	34 (64)	49 (89)
Sustained	11 (21)	5 (9)
Unclear	8 (15)	1 (2)
Event onset		
Abrupt	52 (98)	53 (96)
Insidious	1 (2)	2 (4)
Rare event	46 (87)	48 (87)
Recurrent event (for self-controlled case series only)	-	42 (76)
Rare and/or recurrent event (for self-controlled case series only)	-	55 (100)
All major validity assumptions fulfilled^a^	35 (66)	48 (87)
Minor assumptions:		
Same opportunity of exposure^b^	34/35 (97)	-
Time trend in exposure		
Yes	8/35 (23)	-
*In case of exposure time trend, the design is adapted*	*5/8 (63)*	
No	5/35 (14)	-
Unclear	22/35 (63)	-
Absence of exposure time trend (or design adapted)	32/35 (91)	
Independence of consecutive events		
Yes	-	5/48 (10)
No	-	17/48 (35)
*For non-independent recurrences, the design is adapted*		*16/17 (94)*
Unclear	-	14/48 (29)
Not applicable (for non-recurrent events)	-	12/48 (25)
Independence of consecutive events when recurrent (or design-adapted)^c^	**-**	47/48 (98)
Event-independent exposure		
Yes	-	5/48 (10)
No	-	38/48 (79)
*For non-event-independent recurrences, the design is adapted*		*32/38 (84)*
Unclear	-	5/48 (10)
Event-independent exposure (or design-adapted)^d^	**-**	42/48 (88)
The event affects the short-term mortality probability (censoring of the observation period)	-	
Yes	-	12/48 (25)
*For event-dependent censoring, the design is adapted*		*4/12 (33)*
No	-	36/48 (75)
Absence of censoring the observation period (or design-adapted)^e^	**-**	40/48 (83)
All minor validity assumptions fulfilled for use of a self-controlled design (among articles with valid major assumptions)^f^	31/35 (89)	34/48 (71)
All major and minor validity assumptions fulfilled for use of a self-controlled design (among all articles)	31/53 (58)	34/55 (62)

Data are reported as n (%)

^a^For case-crossover design, a transient or intermittent exposure, an abrupt onset event, and a rare event. For self-controlled case series, a transient or intermittent exposure, an abrupt onset event, and a rare and/or recurrent event. Unclear exposures were considered intermittent

^b^Same opportunity of exposure during case and control time periods

^c^This assumption is fulfilled for studies examining non-recurrent events, recurrent events with independence between consecutive events, or non-independent recurrences when the design is adapted, or recurrent events for which independence is unclear

^d^This assumption is fulfilled for studies examining exposure whose probability is not affected by previous events, with an adapted design when probability of exposure is affected by a previous event, or with unclear event-independent exposures

^e^This assumption is fulfilled for studies examining outcomes that do not censor the observation period by affecting the short-term mortality probability or with an adapted design when the observation period can be censored by the outcome

^f^For case-crossover design, the opportunity for exposure should be the same during the case and control time periods, and there should not be any time trend in exposure. For self-controlled case series, two consecutive events should be independent if they are recurrent; the probability of further exposure should not be affected by a previous event; and the event should not affect the short-term mortality probability nor censor the observation period. In case of violation of one of these assumptions, the design should be adapted

Considering the quality of reporting (Table 3), the assumptions for use of a self-controlled design were reported in 53 (50%) papers (at least one major assumption reported in 41, at least one minor assumption in 8, and both major and minor assumptions reported in 4). In 9 papers stating that the use of a self-controlled design was adequate with regard to major assumptions, the studied exposure was actually sustained. Overall, 94 (88%) papers reported a rationale for using a self-controlled design (i.e., to account for confounding factors that do not change over time). The definition of the control periods was unreported in 3 (6%) papers describing a case-crossover design and 6 (11%) a self-controlled case series. The model used was reported in 93% of articles and was appropriate in 100% of these. The sample size or post-hoc power calculation was reported in 12% of articles. The results were always displayed with a measure of variability, but in 37 (66%) of the self-controlled case series, the person-time in each period was not reported. Two thirds of articles reported adjusted estimates (with or without unadjusted estimates). Sensitivity analyses were described in half of the articles and well reported. Three articles reported all the recommended quality items.

**Table 3:** Quality of reporting

Required items to be reported in the	
Method section	
Assumptions for the use of a self-controlled design (whether the setting is valid for the design implementation, with regard to major assumptions)	53 (50)
Case and control period definition (including number and duration) for case-crossover studies	50/53 (94)
Risk and control period definition (including number and duration) for self-controlled case-series	49/55 (89)
Sensitivity analyses conducted (varying periods duration)	54 (50)
Statistical model	99 (93)
*With the appropriate model* ^a^	*99/99 (100)*
Effect estimator	107 (100)
Sample size or power calculation	13 (12)
Result section	
Appropriate effect estimator with a measure of variability	102 (95)
Person-time in the different periods (for self-controlled case series)	18/55 (33)
Estimate displayed	
Unadjusted effect estimators	36 (34)
Adjusted effect estimators	38 (36)
Both unadjusted and adjusted effect estimators	33 (31)

Data are reported as n (%)

^a^Conditional logistic regression or conditional Poisson regression model for case-crossover studies, conditional Poisson regression model, Cox’s stratified proportional hazards model or conditional logistic regression model for self-controlled case series

## Discussion

In this review of articles, we quantified the appropriate use of self-controlled designs in pharmacoepidemiology involving electronic healthcare databases in terms of major and minor validity assumptions for the use of the designs. We focused on studies involving medical databases, in which self-controlled designs are particularly useful to adjust for time-invariant confounders that may not be collected. Self-controlled designs were not appropriately used in 34% and 13% of the articles we reviewed that described a case-crossover or self-controlled case series design, respectively. We encourage better use of these designs for situations in which major validity assumptions are fulfilled (i.e., for which they are recommended), accounting for situations for which the design can be adapted.

Our study updated the Nordmann et al. review [6] and is the first systematic review exploring the appropriate use of self-controlled designs in pharmacoepidemiology involving electronic healthcare databases, in terms of major and minor validity assumptions, in accordance with recent recommendations. The fulfilment of major assumptions is the minimum requirement for self-controlled designs to be valid, as they can be superior to designs with comparison groups in such situations (more powered and less biased) [5]. Moreover, recent recommendations state that the self-controlled designs should be preferred to designs with comparison groups in studies performed on healthcare databases, when key validity assumptions are fulfilled [4]. For articles that did not fulfil all of the major validity assumptions, it was essentially due to the study of sustained exposure (e.g., antihypertensive drugs or prophylaxis for cardiovascular events) or events with an insidious onset (depression or chronic fatigue). The intermittency of exposure is a requirement for both case-crossover and self-controlled case series to ensure that the number of patients with varying exposure statuses is not too small [4]. The acuteness of the event onset is a validity assumption that reduces the likelihood of misclassification bias [32]. However, some have proposed an adaptation of the case-crossover design for studying prolonged exposures and insidious-onset outcomes [28] and an adaptation of the self-controlled case series (towards the end of our observation period) for studying cumulative exposure [33]. The design adaptation Wang et al. proposed consists of lengthening exposure assessment windows [28]. Despite these adaptations, self-controlled designs are usually less powered than between-person comparisons when studying sustained exposures, because discordant pairs seldom arise when exposures are actually sustained and the observation period is short as compared to the risk and control period durations. Thus, both case-crossover and self-controlled case series designs would fail: the former because of a too-small number of discordant pairs and the latter because cases would be seldom unexposed. Hence, self-controlled designs are not recommended for situations of sustained exposure because they could lead to a loss of power [4]. In fact, few statistical power (or sample size) calculations were carried out in the included studies so the impact of studying long-term exposures when there is a low probability of switching is uncertain. In addition, lengthening exposure assessment windows increases the risk of bias due to within-person time-varying confounders (the absence of which is the main advantage of using self-controlled designs). Even if lengthening exposure assessment windows reduces misclassification bias, it does not answer the issue of reverse causality that could arise when an exposure occurs after the true time of outcome onset, thereby leading to a spurious association. More generally, failure to meet important assumptions of self-controlled designs has been associated with increased risk of discrepant results between case-only and cohort-based approaches (which can occur even in the absence of unmeasured confounders) [30].

For all these reasons, we considered that studies involving a self-controlled design were invalid in situations of sustained exposure when there was a small probability of switching between exposure status within the observation window or when the outcome has an insidious or delayed onset or results from a cumulative effect. Of note, we classified drugs that are usually used chronically as “intermittent exposures” when a high opportunity for a switch from exposed to unexposed status (or vice versa) can be assumed over the observation period. Several examples can be cited: methylphenidate in children with attention-deficit/hyperactivity disorder (the treatment is usually discontinued during holidays) and palivizumab, an anti-respiratory syncytial virus (RSV) monoclonal antibody for prophylaxis of severe lower respiratory tract infection in children (usually administered during the high-risk season of RSV infection).

In a sensitivity analysis, considering studies examining a sustained exposure as appropriate (without accounting for the probability of switching exposure status), 45 (85%) case-crossover and 53 (96%) self-controlled case series studies fulfilled all major assumptions. In this analysis, the most frequent validity threats were insidious or common events.

In terms of event frequency, we considered rare and/or recurrent events as appropriate for self-controlled case series and only rare events for case-crossover designs. However, in situations when the event is both non-recurrent and non-rare, a self-controlled design can still be used. Nevertheless, this use would imply that the number of strata (here, the number of cases) would increase but not their size (here, the number of periods within the same patient), thereby leading to poor estimation of the variance when using stratified models. Therefore, we considered the rare or recurrent event as a major assumption. Moreover, in the study of Pouwels et al., the rareness of the outcome was a factor associated with fewer discrepancies [30].

We found that minor assumptions were most often valid when major ones were valid. As a reminder, those assumptions were considered minor because the design can be adapted if they are not fulfilled, which allows for a self-controlled design. A small proportion of the self-controlled designs, 18 (16%), could have been improved by applying those adaptations. For instance, 3 studies with a case-crossover design did not adjust for a time trend in exposure, even though the paper clearly stated that such a trend existed. It has been shown that lack of adjustment for exposure time-trends in case-crossover studies led to biased estimations [14, 25, 34], and hence several extensions of the case-crossover design have been developed to take into account a temporal trend in exposure [14, 15, 24, 25]. Of note, in 22 additional papers from our systematic review, the existence of an exposure time-trend was not discussed by the authors nor could be assessed from the reported information, but we still considered them appropriate. Thus, the proportion of case-crossover studies that could have used the design more adequately may be underestimated. Researchers must keep in mind that the exploration and reporting of such a trend in case-crossover studies is crucial for design validity. Concerning the event-independent exposure assumption, we found that it was fulfilled in 88% of articles involving a self-controlled case series. A simulation study reported that relative incidence is almost always overestimated when the event-independent exposure assumption is violated in self-controlled case series studies (except for the situation of extreme dependence), but the bias is corrected when the design is adapted [35]. The corresponding methodological developments were published in the late 2000s [11, 16], perhaps too recently to be applied in the studies we reviewed.

Of note, a simulation study explored the validity of the case-time-control design in situations of within-individual exposure dependency over several control periods but showed that the method is robust to deviation of this assumption [36]. We did not explore this assumption the studies included in our review.

The previous systematic review by Nordmann et al. reported the validity assumptions of self-controlled designs in pharmacoepidemiology between 1995 and 2010 (before the development of the previously cited recommendations) [6]. The authors reported an inappropriate use of self-controlled designs: validity assumptions were not fulfilled for 76% of the articles describing a case-crossover design and 60% self-controlled case series. Concerning major assumptions, our review, which covered healthcare database studies published recently, shows that these data have improved. Moreover, major and minor validity assumptions were not distinguished in the Nordmann et al. review. Nevertheless, we found the same main reasons for the inappropriate use of these designs (i.e., the study of sustained exposure and the absence of considering exposure time-trend).

Self-controlled designs can control for intra-individual time-invariant confounders. Many design extensions that weaken the validity assumptions have been developed, and these designs still are under development, such as for the study of multiple exposures [37], or the study of recurrent events when recurrences are not independent [27]. However, the designs are subject to several biases (e.g., residual confounding due to unmeasured within-person time-varying factors or misclassification of exposure [32]). Moreover, self-controlled designs explore the triggers that precede abrupt-onset events, and answer the questions “Why now?” or “What happened just before?”, which is slightly different from the question raised with between-person comparisons (“Why me?”) [38]. Nevertheless, they are complementary to cohort-based approaches [4], and both designs should be applied, especially when one or more assumptions are not fulfilled [30].

Regarding the quality of reporting, we found 9 studies examining sustained exposures (e.g., antihypertensive treatments or low-dose aspirin for secondary prevention of cardiovascular events), which indicates the reporting of the design being appropriate to study abrupt-onset outcome and transient drug exposure. This high number underlines that authors and reviewers should be aware of the design’s validity assumptions, recommendations for use and the need to report validity assumptions fulfilled (or not). Indeed, the minor assumptions were rarely reported in the papers we reviewed. In addition, the sample size or power calculation was rarely reported, with no improvement compared to a previous review [6]. However, studies involving electronic healthcare databases usually have very large sample sizes and perhaps the sample size calculation is not needed in this context, because the sample size cannot be chosen. However post-hoc power calculation in the database study sample is important to decide which healthcare database should be used and to interpret the absence of a statistical association, especially in the context of a very rare event. Post-hoc power calculation indicates how easily an effect that is fixed a priori can be shown (accounting for the observed number of patients/cases and the observed variability). Even if confidence intervals (which represent how accurate the results are estimated) are reported, the power calculation is more related to the number of observed events, and is information that is easier for the reader to understand: in case of non-significant associations, power can be quite difficult to interpret on the basis of the sole confidence interval.

With respect to other elements of reporting quality, a measure of variability of the estimate was always reported, which we considered adequate if the number and duration of different periods were also reported. The effect estimator was not always appropriately reported, but reported statistical models were all considered as appropriate. Valid models other than the conditional logistic regression or the conditional Poisson regression can still be applied, such as the Cox stratified proportional-hazards model for case-crossover studies and the Cox stratified or conditional logistic regression models for self-controlled case series [30], even if unusual. However, it has been shown that in case-crossover studies, for instance, conditional logistic regression model and conditional Poisson model give identical estimates [29]. In case-crossover studies, times to event are the same for case and control periods (because periods are defined similarly within strata) and even if a stratified Cox model can be used, it does not assess and compare a time to event. Therefore, the estimator computed (even if called a “hazard ratio”) still represents an “odds ratio” and should be interpreted as such. One third of the included papers reported both adjusted and unadjusted estimates, which is quite low when they allow for assessing the importance of bias. Nordmann et al. recommended that the number of discordant pairs in case-crossover designs (i.e., number of patients who crossed from unexposed in the control period to exposed in the case period, or vice versa [8]), or the count of events in the different time periods for self-controlled case series should be reported [6]. We considered that these items need to be reported, except if a measure of variability of the estimate is reported along with a clear description of the number and duration of different periods. However, only a small number of self-controlled case series reported the duration of control period along with the person-times in risk and control periods, but this was appropriately reported for case-crossover studies. Consequently, the quality of reporting in self-controlled studies can still be improved, in accordance with the recommendations provided by Nordmann et al. [6].

Our study has some limitations. First, a potential paper selection bias could exist, which we tried to limit with a comprehensive literature search using keywords, title and abstract terms, and few limits, that were already used in previous systematic reviews [6, 30, 39]. Moreover, the definition of abrupt versus insidious onset of the event is somewhat subjective, as is transient versus sustained exposure, or some minor assumptions for the self-controlled case series. We tried to limit this issue by consensus. Some strengths of this study are worth noting. We focused on studies involving medical databases because of a growing interest in the use of “big data” in healthcare research [40]. We updated Nordmann et al. review [6] up to 2014, and to ensure that no indexing issue in PubMed can be suspected, we updated our literature search in July 2016.

## Conclusion

Self-controlled designs have many advantages, including their ability to inherently adjust for time-invariant factors, which is important when using electronic healthcare databases, where some confounding factors are usually not collected or nor available. We found that in terms of fulfilling the major assumptions of the designs, the designs for one-third of the case-crossover studies and less than one-fifth of self-controlled case series we reviewed were not appropriately used in these pharmacoepidemiology studies involving electronic healthcare databases. We encourage a better justification of the design validity in terms of major and minor assumptions in accordance with recommendations for their use and more accurate reporting of self-controlled case series. Addressing these issues will contribute to a wiser use of these self-controlled designs, which is advantageous for pharmacoepidemiology involving large healthcare databases.

## Additional file

Additional file 1: S1. MEDLINE search algorithm. **S2.** Standardized extraction form and specific validity assumptions. **S3.** Articles included in the review. **S4.** Validity assumptions for each article describing a case-crossover (4a) or a self-controlled case-series design (4b). **S5.** Validity assumptions for the use of a self-controlled design. Sensitivity analysis when unclear assumptions are considered invalid. **S6.** Validity assumptions for the use of a self-controlled design. Sensitivity analysis after excluding articles for the same series. (DOC 797 kb)

## Abbreviations

PRISMA: Preferred reporting items for systematic reviews and meta-analyses
RSV: Respiratory syncytial virus
STROBE: Strengthening the reporting of observational studies in epidemiology

## Footnotes

1 In some articles of self-controlled case series, the risk period was defined arbitrarily in the same way for all patients without accounting for the actual duration of prescription. For drugs used chronically, this situation can result in misclassification bias, because the subject is considered unexposed when a prescription period was still ongoing. Such exposures were considered sustained and the design was considered as inappropriate in these situations. In contrast, when risk period was equal to the prescription period and when the observation period was large enough to observe a switch in exposure status, we considered the exposure as transient even if it was used chronically
