# Supplementary material for: Self-controlled designs in pharmacoepidemiology involving electronic healthcare databases: a systematic review
Source: BMC Med Res Methodol. 2017 Feb 8;17:25. doi: 10.1186/s12874-016-0278-0 (PMC5299667; doi:10.1186/s12874-016-0278-0)
Supplement: Additional file 1: S1. — MEDLINE search algorithm. S2. Standardized extraction form and specific validity assumptions. S3. Articles included in the review. S4. Validity assumptions for each article describing a case-crossover (4a) or a self-controlled case-series design (4b). S5. Validity assumptions for the use of a self-controlled design. Sensitivity analysis when unclear assumptions are considered invalid. S6. Validity assumptions for the use of a self-controlled design. Sensitivity analysis after excluding articles for the same series. (DOC 797 kb) [file 12874_2016_278_MOESM1_ESM.doc]

**Additional file 1**

[Supplementary Material S1: MEDLINE search algorithm 1](#__RefHeading___Toc467676846)

[Supplementary Material S2: Standardized extraction form and specific validity assumptions 3](#__RefHeading___Toc467676847)

[Supplementary Material S3: Articles included in the review 16](#__RefHeading___Toc467676848)

[Supplementary MaterialS4a: Validity assumptions for each article describing a case-crossover design 22](#__RefHeading___Toc467676849)

[Supplementary Material S4b: Validity assumptions for each article describing a self-controlled case-series design 25](#__RefHeading___Toc467676850)

[Supplementary Material S5: Validity assumptions for the use of a self-controlled design. Sensitivity analysis when unclear assumptions are considered invalid. 28](#__RefHeading___Toc467676851)

[Supplementary Material S6. Validity assumptions for the use of a self-controlled design. Sensitivity analysis after excluding articles for the same series. 29](#__RefHeading___Toc467676852)

# Supplementary Material S1: MEDLINE search algorithm

*Terms to identify self-controlled designs (excluding non-original articles, reviews or trials)*

("case-crossover"[Tiab] OR "case crossover"[Tiab] OR "case cross-over"[Tiab] OR "case cross over"[Tiab]

OR "case-time-control"[Tiab] OR "case time-control"[Tiab] OR "case-time control"[Tiab] OR "case time control"[Tiab] OR "self-controlled case series"[Tiab] OR "self controlled case series"[Tiab] OR "self controlled case-series"[Tiab] OR "self-controlled case-series"[Tiab] OR "case series analysis"[All Fields] OR ("self control"[All Fields] AND "case series"[All Fields]) OR "case-only"[Tiab] OR "case only"[Tiab] OR "self-controlled design"[Tiab] OR "self controlled design"[Tiab])

NOT

("review"[Publication Type] OR "Meta-Analysis"[Publication Type] OR "case reports"[Publication Type] OR "Editorial"[Publication Type] OR "Letter"[Publication Type] OR "Randomized Controlled Trial"[Publication Type] OR "Clinical Trial, Phase I"[Publication Type] OR "Clinical Trial, Phase II"[Publication Type] OR "Clinical Trial, Phase III"[Publication Type] OR "Comment"[Publication Type] OR "Controlled Clinical Trial"[Publication Type] OR "double-blind"[Tiab] OR "placebo-controlled"[Tiab] OR "pilot study"[Tiab] OR "pilot projects"[Tiab])

Limits : Humans, English, French, 1^st^ January 2011 – 31^st^ december 2014

# Supplementary Material S2: Standardized extraction form and specific validity assumptions

**GENERAL DATA**

**Article Number** ……… **Reader** ………………… **Publication date** (dd/mm/aaaa) …………………...… **Journal** **(abrv)**…………………

**Title**……………………………..……………………………..……………………………..……………………………..……………………………

**First author name** ……………………………..…………………………….…….…….…….…….…….…….…….…….…….…….…….…….….

**Funding** **Conflicts of interest disclosed** _1_ Yes _0_ No

1 Public If yes, conflict of interest reported _1_ Yes _0_ No

2 Private

3 Both public and private

4 No funding

5 NR[[1]](#footnote-2)

6 Other…………………………………………………………………………………………………………………………………………………

**Medical area of the study population/patients**[[2]](#footnote-3)  **Medical area of the exposure Medical area of the outcome**

1 Psychiatry _1_ Psychiatry _1_ Psychiatry

2 Immunology/vaccine _2_ Immunology/vaccine _2_ Immunology/vaccine

3 Pediatry _3_ Pediatry _3_ Pediatry

4 Cardiovasular _4_ Cardiovasular _4_ Cardiovasular

5 Rheumatology _5_ Rheumatology _5_ Rheumatology

6 Neurology/neurovasc _6_ Neurology/neurovasc _6_ Neurology/neurovasc

7 Infectiology _7_ Infectiology _7_ Infectiology

8 Obstetric _8_ Obstetric _8_ Obstetric

9 Pneumology _9_ Pneumology _9_ Pneumology

10 Gastro-enterology _10_ Gastro-enterology _10_ Gastro-enterology

11 Oncology _11_ Oncology _11_ Oncology

12 Not specified / population based _12_ Not specified / population based _12_ Not specified / population based

13 Other …………………………………… _13_ Other …………………… _13_ Other ……………………………………

**Study period** (from the 1^st^ prescription recorded to the last event observed)

Starting date (dd/mm/aaaa) ………………… Ending date (dd/mm/aaaa) ………………… Length …… _1_ h _2_ d _3_ w _4_ m _5_ y _6_ NR

**Size of the eligible population in the database**[[3]](#footnote-4) (rounded to the nearest thousand) ……………………………..  NR

**DATABASES** *(to be filled for all the articles)*

**Exposure data collection**:

A database is used for the exposure assessment _1_ Yes _0_ No _2_ NR

**Date of implementation** (dd/mm/aaaa): ……………………  NR

**Geographic area covered**: …………………………………………………………………………………………………………………………

**Type of the database**:

1 Administrative database (prescription reimbursement) _6_ Interview / Telephone call

2 Institutional/ hospital records (electronic medical records) _7_ Self-report questionnaire or diary

3 Institutional/ hospital records (paper medical records) _8_ Previous cohort study

4 Primary care database _9_ Others: ….…….…….…….…….…….…….…….……………………...

5 Pharmacy records (prescription delivery)

**Name of the database:**

1 Dutch Health Care Insurance Board (NL) _12_ Ontario health insurance database (CA)

2 EGB / SNIIR-AM (FR) _13_ Pedianet

3 GPRD _14_ PharMetrics (USA)

4 GP Research Network _15_ Pharmo

5 Group Health (USA) _16_ PDNJ

6 Health Search Database _17_ RamQ database (Quebec)

7 Kaiser Permanente (USA) _18_ Saskatchewan databases (Canada)

8 Medicaid / Medicare (USA) _19_ Tayside Medicine Monitoring Unit (MEMO, Scotland)

9 Mediplus Patient Database _20_ VA database (USA)

10 National health insurance database (Taiwan) _21_ Other: …….…….…….…….…….…….…….…….…….……….………

11 OPED

**Size of the database**

The size of the database is reported _1_ Yes _0_ No

If yes: size of the database (rounded to the nearest thousand): ……………… Date of last update: ………………………………………………

**Special population covered[[4]](#footnote-5)** _1_ Yes _0_ No. Specify: ………………………………………………………………………………………………

**Data collected for the study**

Drugs: Classification code: Other data:

1 prescription _1_ ATC Quantity _1_ Yes _0_ No _2_ NR

2 delivery _2_ National drug code (FDA) Dosage regimen _1_ Yes _0_ No _2_ NR

3 reimbursement _3_ Other: ……………………………… Strength _1_ Yes _0_ No _2_ NR

4 other: …………………………… _4_ NR Treatament Indication _1_ Yes _0_ No _2_ NR

5 NR

OTC drugs collected[[5]](#footnote-6): _1_ Yes _0_ No _2_ NR.

Source of OTC collection _1_ Collected from the database _2_ collected from an other source: ……………………………………………

Inpatient drugs collected: _1_ Yes _0_ No _2_ NR.

Source of Inpatient drugs collection _1_ Collected from the database _2_ collected from an other source: …………………………………

**Validity of data collected:**

The validity of recorded prescriptions is referenced _1_ Yes _0_ No ; or is reported _1_ Yes _0_ No. Specify ……………………………………

**Event data collection**:

A database is used for the event assessment _1_ Yes _0_ No _2_ NR

The database used for the event’s assessment is the same as for the exposure _1_ Yes _0_ No.

If not, **date of implementation** (dd/mm/aaaa): ……………………  NR

**Geographic area covered**: ……………………………………………………………………………………………………………………………

**Type of the database**:

1 Administrative database (prescription reimbursement) _6_ Interview / Telephone call

2 Institutional/ hospital records (electronic medical records) _7_ Self-report questionnaire or diary

3 Institutional/ hospital records (paper medical records) _8_ Previous cohort study

4 Primary care database _9_ Others: ….…….…….…….…….…….…….…….……………………...

5 Pharmacy records (prescription delivery)

**Name of the database:**

1 Dutch Health Care Insurance Board (NL) _12_ Ontario health insurance database (CA)

2 EGB / SNIIR-AM (FR) _13_ Pedianet

3 GPRD _14_ PharMetrics (USA)

4 GP Research Network _15_ Pharmo

5 Group Health (USA) _16_ PDNJ

6 Health Search Database _17_ RamQ database (Quebec)

7 Kaiser Permanente (USA) _18_ Saskatchewan databases (Canada)

8 Medicaid / Medicare (USA) _19_ Tayside Medicine Monitoring Unit (MEMO, Scotland)

9 Mediplus Patient Database _20_ VA database (USA)

10 National health insurance database (Taiwan) _21_ Other: …….…….…….…….…….…….…….…….…….……….………

11 OPED

**Size of the database**

The size of the database is reported _1_ Yes _0_ No.

If yes: size of the database (rounded to the nearest thousand): ……………… Date of last update: ………………………………………………

**Special population covered[[6]](#footnote-7)** _1_ Yes _0_ No. Specify: ………………………………………………………………………………………………

**Data collected for the study**

Diagnoses and procedures reported : _1_ Yes _0_ No Classification code reported : _1_ Yes _0_ No

inpatient records _1_ Yes _0_ No ICD-9 _1_ Yes _0_ No

outpatient records _1_ Yes _0_ No ICD-10 _1_ Yes _0_ No

primary care record_1_ Yes _0_ No READ / OXMIS _1_ Yes _0_ No

specialist referral _1_ Yes _0_ No Other _1_ Yes _0_ No ………………………………

other _1_ Yes _0_ No ………………………………………………………………………………

**Validity of data collected:**

The validity of recorded diagnoses is referenced _1_ Yes _0_ No ; or is reported _1_ Yes _0_ No. Specify ………………………………………

**Confonding data collection**:

A database is used for the confounding data assessment _1_ Yes _0_ No

The database used for the confounding data assessment is the same as:

for the exposure _1_ Yes _0_ No,

for the event _1_ Yes _0_ No.

If not, **date of implementation** (dd/mm/aaaa): ……………………  NR

**Geographic area covered**: ……………………………………………………………………………………………………………………………

If not, **type of the database**:

1 Administrative database (prescription reimbursement) _6_ Interview / Telephone call

2 Institutional/ hospital records (electronic medical records) _7_ Self-report questionnaire or diary

3 Institutional/ hospital records (paper medical records) _8_ Previous cohort study

4 Primary care database _9_ Others: ….…….…….…….…….…….…….…….……………………...

5 Pharmacy records (prescription delivery)

**Name of the database:**

1 Dutch Health Care Insurance Board (NL) _12_ Ontario health insurance database (CA)

2 EGB / SNIIR-AM (FR) _13_ Pedianet

3 GPRD _14_ PharMetrics (USA)

4 GP Research Network _15_ Pharmo

5 Group Health (USA) _16_ PDNJ

6 Health Search Database _17_ RamQ database (Quebec)

7 Kaiser Permanente (USA) _18_ Saskatchewan databases (Canada)

8 Medicaid / Medicare (USA) _19_ Tayside Medicine Monitoring Unit (MEMO, Scotland)

9 Mediplus Patient Database _20_ VA database (USA)

10 National health insurance database (Taiwan) _21_ Other: …….…….…….…….…….…….…….…….…….……….………

11 OPED

**Size of the database**

The size of the database is reported _1_ Yes _0_ No.

If yes: size of the database (rounded to the nearest thousand): ……………… Date of last update: ………………………………………………

**Special population covered** _1_ Yes _0_ No. Specify: …………………………………………………………………………………………………

**Data collected for the study**

**Demographics: Behaviour: Clinical informations:**

Age _1_ Yes _0_ No [[7]](#footnote-8) _2_ NR Alcohol consumption _1_ Yes _0_ No _2_ NR BMI _1_ Yes _0_ No _2_ NR

Gender _1_ Yes _0_ No _2_ NR Smoking status _1_ Yes _0_ No _2_ NR Co-morbidities _1_ Yes _0_ No _2_ NR

Live place _1_ Yes _0_ No _2_ NR Physical exercise _1_ Yes _0_ No _2_ NR Family history _1_ Yes _0_ No _2_ NR

Ethnicity _1_ Yes _0_ No _2_ NR Diet _1_ Yes _0_ No _2_ NR Laboratory tests _1_ Yes _0_ No _2_ NR

Socioeco status _1_ Yes _0_ No _2_ NR X-ray exams _1_ Yes _0_ No _2_ NR

Ttt Indication _1_ Yes _0_ No _2_ NR

Other therapy _1_ Yes _0_ No _2_ NR

**Validity of data collected:**

The validity of recorded data is referenced _1_ Yes _0_ No ; or is reported _1_ Yes _0_ No. Specify ………………………………………………

**Advantages and disadvantages**

Advantages and interests of using databases are reported by the authors _1_ Yes _0_ No

Disadvantages or limits of using databases are reported by the authors _1_ Yes _0_ No

Specify: ………………………………………………………………………………………………………………………………………………… ……………………………………………………………………………………………………………………………………………………………

……………………………………………………………………………………………………………………………………………………………

*The remaining form to be filled only for the first 102 articles.*

**EXPOSITION DATA**

**Number of distinct studied classes of molecules** (only consider the primary analysis) ………………………………………………………………

**Classes of molecule:** ………………………… ………………………… ………………………… …………………………

………………………… ………………………… ………………………… …………………………

The exposure is collected/measured prospectively[[8]](#footnote-9) _1_ Yes _0_ No _2_ NR

The exposure is measured independently of the case status[[9]](#footnote-10)  1 Yes _0_ No _2_ NR

The exposure is measured in the same way during case and control (or exposed/unexposed) periods/patients _1_ Yes _0_ No _2_ NR

**Definition of the exposed group as reported in the article:** (only consider the primary analysis) …………………………………………………

……………………………………………………………………………………………………………………………………………………………

The exposure definition uses a proxy _1_ Yes _0_ No _2_ NR If yes, which one?

1 Prescription

2 Delivery

3 Reimbursement

4 Other …….…….…………………………………………………………

The exposure definition require a given number (>1) of drug taking: _1_ Yes _0_ No _2_ NR If yes, how many? ……………………………...

**Classification of the exposure:** (only consider the primary analysis. If several classifications, consider they are sensitivity analyses)

Dichotomous variable (yes/no) _1_ Yes _0_ No _2_ NR

Qualitative ordinal variable (past, recent, current, non user _1_ Yes _0_ No _2_ NR

short term, long term, non user)

Qualitative nominal variable (regular, non regular user) _1_ Yes _0_ No _2_ NR

Quantitative (cumulative dose) _1_ Yes _0_ No _2_ NR

Quantitative (cumulative length of exposure) _1_ Yes _0_ No _2_ NR

Time dependant definition (person-time) _1_ Yes _0_ No _2_ NR

**Characteristics of exposure** (only consider the primary analysis)

Average prevalence of the exposure in the study population[[10]](#footnote-11) is reported in the article _1_ Yes _0_ No _2_ NA[[11]](#footnote-12)

If yes : specify…….…….…….…….

Type of exposure (as reported): Type of exposure (to your knowledge):

1 one shot (e.g. vaccine) _1_ one shot (e.g. vaccine)

2 transient/intermittent[[12]](#footnote-13) _2_ transient/intermittent

3 acute[[13]](#footnote-14) (few days) _3_ acute (few days)

4 sustained (>1 month) _4_ sustained (>1 month)

5 NR _5_ DK

Time trend exposure in the eligible population is reported[[14]](#footnote-15): _1_ Yes _0_ No

If yes, the time trend is controlled for _1_ Yes _0_ No _2_ NR

Specify: ….…….…….…….…….…….…….…….…….….…….…….…….…….…….…….…….…….….…….…….…….…….…….…….……

The probability of exposure is independent of the occurrence of the outcome[[15]](#footnote-16) _1_ Yes _0_ No _2_ NR

The exposure can be the consequence of the event[[16]](#footnote-17) _1_ Yes _0_ No _2_ NR

**HEALTH OUTCOME**

**Definition of the outcome as reported in the article:** (only consider the primary analysis)….…….…….…….…….…….…….…….…….…….

….…….…….…….…….…….…….…….…….…….….…….…….…….…….…….…….…….…….…….….…….…….…….…….…….…….…

**Event data collection**:

The outcome was collected prospectively _1_ Yes _0_ No _2_ NR

The outcome is collected independently of the exposure status[[17]](#footnote-18) _1_ Yes _0_ No _2_ NR

The outcome is measured in the same way in case and control periods/patients, or in exposed/unexposed periods/patients _1_ Yes _0_ No _2_ NR

**Definition of the event** (only consider the primary analysis)

The event definition is clearly reported (e.g. diagnostic criteria are reported[[18]](#footnote-19)) _1_ Yes _0_ No

The event definition uses a proxy[[19]](#footnote-20) _1_ Yes _0_ No _2_ Partly _3_ NR

If yes, which one…………………………………………………………………………………………………………………………………………

**Characteristic of the event** (only consider the primary analysis)

Average prevalence of the event in the exposed group, as reported in the article………………….  NR  NA (case-control)

Average prevalence of the event in the unexposed group, as reported in the article……………….  NR  NA (case-control)

Average overall prevalence of the event, as reported in the article[[20]](#footnote-21)………………………………  NR

**Type of event:** ………………….………………….

Recurrence: Onset: Reversibility: Duration:

1 recurrent _1_ acute onset _1_ reversible[[21]](#footnote-22) _1_ acute

2 unique _2_ insidious onset _2_ irreversible[[22]](#footnote-23) _2_ chronic

3 partially irreversible[[23]](#footnote-24)

**Type of event:** ………………….………………….

Recurrence: Onset: Reversibility: Duration:

1 recurrent _1_ acute onset _1_ reversible _1_ acute

2 unique _2_ insidious onset _2_ irreversible _2_ chronic

3 partially irreversible

**Type of event:** ………………….………………….

Recurrence: Onset: Reversibility: Duration:

1 recurrent _1_ acute onset _1_ reversible _1_ acute

2 unique _2_ insidious onset _2_ irreversible _2_ chronic

3 partially irreversible

**Type of event:** ………………….………………….

Recurrence: Onset: Reversibility: Duration:

1 recurrent _1_ acute onset _1_ reversible _1_ acute

2 unique _2_ insidious onset _2_ irreversible _2_ chronic

3 partially irreversible

Time trend in event occurrence is reported[[24]](#footnote-25) : _1_ Yes _0_ No

If yes, the time trend is controlled for _1_ Yes _0_ No _2_ NR. How is it controlled for…….…….…….…….…….…….…….…….…

**EXPOSURE – HEALTH OUTCOME ASSOCIATION (in the study hypotheses on the primary objective)**

The exposure induces potentially short-term risk/effect (as reported in the article) _1_ Yes _0_ No _2_ NR

The exposure induces potentially short-term risk/effect (to your knowledge) _1_ Yes _0_ No _2_ DK

Specify: ….…….…….…….…….…….…….……….…….…….…….…….…….…….…….…….……….…….…….…….…….…….…….…….

The exposure induces effect potentially after an induction time (as reported in the article) _1_ Yes _0_ No _2_ NR

The exposure induces effect potentially after an induction time (to your knowledge) _1_ Yes _0_ No _2_ DK

Specify: ….…….…….…….…….…….…….……….…….…….…….…….…….…….…….…….……….…….…….…….…….…….…….…….

**DESIGN**

**Type of study :**

1 Cohort

2 Case-control

3 Nested case control

4 Case-crossover

5 Case-time control

6 SCCS

7 other: ………………………………………………………………………………………………………………………………………………

**If case-crossover or case-time control or SCCS designs:**

Definition of the control period: ………………………………………………………………………………………………………………………...

Length of the control period: ……………… _1_ h _2_ d _3_ w _4_ m _5_ y _6_ NR

Number of control period per patient: ………………………………  NR  NA (SCCS)

The control period duration is the same for all patients  1 Yes _0_ No  2 NR _3_ NA (SCCS)

Definition of the risk period: ……………………………………………………………………………………………………………………………

Definition is based on:

1 physiological evidence

2 practical issues

3 previous study

4 NR

The risk period begins: Time lapse between the start of risk period and the event/exposure:

1 before the event ……………… _1_ h _2_ d _3_ w _4_ m _5_ y _6_ NR

2 after the exposure ……………… _1_ h _2_ d _3_ w _4_ m _5_ y _6_ NR

Length of the risk period ……………… _1_ h _2_ d _3_ w _4_ m _5_ y _6_ NR

The risk period duration is the same for all patients _1_ Yes _0_ No _2_ NR

**If SCCS** **designs:**

The observation period duration is the same for all patients _1_ Yes _0_ No _2_ NR

**If cohort designs:**

Definition of the non exposed group: ………………………………………………………….…………………….…………………….……………

The groups are matched _1_ Yes _0_ No _2_ NR

If yes, number of matching criteria ………..………..

Specifiy the criteria ……………… ……………… ……………… ……………… ……………… ……………… ………………

The unexposed group uses an active comparator _1_ Yes _0_ No _2_ NR

If yes, which one ………………………………………………………………………………………………………………………………………...

The control molecule is one from the same pharmacological class[[25]](#footnote-26) 1 Yes 0 No 2 NR

**If case-control or nested case-control designs:**

Definition of the control group: …………………………………………………………………………………………………………………………

The groups are matched _1_ Yes _0_ No _2_ NR

If yes, number of matching criteria ………..………..

Specifiy the criteria ……………… ……………… ……………… ……………… ……………… ……………… ………………

The unexposed group uses an active comparator 1 Yes 0 No 2 NR

If yes, which one ………………………………………………………………………………………………………………………………………...

The control molecule is one from the same pharmacological class 1 Yes 0 No 2 NR

**POPULATION**

**Eligibility criteria**

Eligibility criteria are described _1_ Yes _0_ No

Only incident cases are included in the study _1_ Yes _0_ No _2_ NR

Only the incident exposed subjects are included in the study (new user design) _1_ Yes _0_ No

If yes, length of the non-exposed period before inclusion ……………… _1_ h _2_ d _3_ w _4_ m _5_ y _6_ NR

**If cohort or case-control or nested case-control designs:**

Definition of the study entry for cases/exposed: Definition of the cohort entry for controls/unexposed:

1 A calendar date (the same for all) _1_ A calendar date (the same for all)

2 A random date _2_ A random date

3 Date of hospital discharge _3_ Date of hospital discharge

4 Date of diagnostic _4_ Date of diagnostic

5 Date of first prescription _5_ Date of first prescription

6 Date of the given number of prescription _6_ Date of the given number of prescription

7 Other …………………………………………………… _7_ Other …………………………………………………………………………

**Representativeness**

The study population can be considered as representative of the target population[[26]](#footnote-27) _1_ Yes _0_ No

**STATISTICS**

**Sample size**

Calculation of the sample size is reported  1 Yes _0_ No

Statistical model**. Type of statistical model used:** **(only consider the primary analysis)**

1 Logistic

2 Conditional logistic

3 Generalized linear

4 Cox

5 Poisson

6 Mantel-Haenszel

7 Hierarchical

8 Mantel-Haenszel

9 Markov

10 Other **……………………………………………………………………………………………………………………………………………**

**Confounding** variables

Adjustment for confounding variables has been made**_1_ Yes** **_0_ No**

If yes, which factors are considered? Specify the factors that are reported.

1 Socio-demographic factors ……………………………………………………………………………………………………………..

2 Behavioral factors (smoking, diet, physical exercise, etc…) …………………………………………………………………………..

3 Biological factors ……………………………………………………………………………………………………………………….

4 Clinical factors (BMI, comorbidities, family history, past medical history, etc…) ……………………………………………………

5 Other medication ……………………………………………………………………………………………………………………….

6 Other ……………………………………………………………………………………………………………………………………

Confounding factors are estimated via a proxy[[27]](#footnote-28) _1_ Yes _0_ No

Proxy definition: ………………………………………………………………………………………………………………...

Adjustment is made using a propensity score (PS) _1_ Yes _0_ No PS used in the primary analysis _1_ Yes _0_ No[[28]](#footnote-29)

If yes, method used:

1 Adjustment

2 Matching

3 Stratification

4 Inverse probability weight (IPTW)

5 Other ……………………………………………………………………………………………………………………………………

6 NR

Number of patients included in the main analysis with the propensity score ………………  NR

Adjustment is made using an instrumental variable (IV) _1_ Yes _0_ No IV used in the primary analysis _1_ Yes _0_ No

**Switch exposure status**

Some patients switch exposure status over time _1_ Yes _0_ No

If yes, a method to take it into account is reported _1_ Yes _0_ No

How did the authors deal with patients that switch exposure during observation period?

1 Excluded them from the analyses

2 Considered time dependant exposition

3 other ………....…….…….…….…….…….……………………....…….…….…….…….…….……………………....…….…….…….………

Sensitivity analysis

Sensitivity analyses are reported[[29]](#footnote-30) **_1_ Yes** **_0_ No**

Detail sensitivity analyses ……………………………………………………………………………………………………………………………….

……………………………………………………………………………………………………………………………………………………………

Type of sensitivity analyses performed:

**For cohort/case-control studies:**

1 Varied definition of exposure

2 Varied definition of event

3 Varied duration of exposed person-times

4 other ………....…….…….…….…….…….………………....…….…….…….…….…….………………....…….…….…….…….

**For case-crossover/case time-control/SCCS studies:**

1 Varied definition of exposure

2 Varied definition of event

3 Varied number of control period

4 Varied duration of induction period

5 Varied duration of control period

6 Varied duration of risk period

7 other ………....…….…….…….…….…….………………....…….…….…….…….…….………………....…….…….…….…….

The choice of varied parameters for the sensitivity analyses have been justified:  1 Yes _0_ No

**Subgroups** analysis

Subgroup analyses have been conducted  1 Yes _0_ No

If yes, the subgroup analyses were planned in advance[[30]](#footnote-31)  1 Yes _0_ No

Type of subgroup analyses performed:

Complete case analysis _1_ Yes _0_ No

Other restriction of the population _1_ Yes _0_ No ……….……………………………….……………………………….…………………………..

Other subgroup _1_ Yes _0_ No ……….……………………………….……………………………….………………………………………………

**RESULTS**

Number of missing data is reported _1_ Yes _0_ No

Reasons of non participation[[31]](#footnote-32) are reported  1 Yes _0_ No

A dose-response gradient[[32]](#footnote-33) has been estimated  1 Yes _0_ No

A test for trend has been used _1_ Yes _0_ No

**Main results** (only consider the primary analysis)

The estimator of the excess of risk is:

1 HR

2 RR

3 OR

4 Relative Incidence

5 Incidence Rate Ratio

6 Prevalence rate ratio

7 Excess Risk

8 Absolute risk

9 other …….…….…….…….…….…….…….…….…….…….…….…….…….…….…….…….…….…….…….…….…….…….…….………

**One main result identified**  **1 Yes** **_0_ No**

If Yes:

**If case-crossover study / case-time control study / SCCS: (only consider the primary analysis)**

Number of cases: …….…….

Number of exposed case[[33]](#footnote-34): …….…….

Number of unexposed case[[34]](#footnote-35): …….…….

If case-time-control: in the control group, natural time trend in drug utilization OR = …….…….  NR

Association results:  NR

Unadjusted = …….…….…….……. 95% IC …….…….…….…….

Adjusted = …….…….…….……. 95% IC …….…….…….…….

**If case-control study:** **(only consider the primary analysis)**

Number of cases: …….…….

Number of controls …….…….

**Association results:**  **NR**

Unadjusted = …….…….…….……. 95% IC …….…….…….…….

Adjusted = …….…….…….……. 95% IC …….…….…….…….

**If cohort study: (only consider the primary analysis)**

Number of exposed patients: …….…….

Number of non exposed patients: …….…….

Association results:  NR

Unadjusted = …….…….…….……. 95% IC …….…….…….…….

Adjusted = …….…….…….……. 95% IC …….…….…….…….

**If other design:** **(only consider the primary analysis)**

Association results:  NR

Unadjusted = …….…….…….……. 95% IC …….…….…….…….

Adjusted = …….…….…….……. 95% IC …….…….…….…….

If more than one main result identified:

Type of effect observed:

1 the drug is a risk factor

2 the drug is a protective factor

3 the drug is either a risk or protective factor (depending on the outcome or the exposition)

4 the drug has no effect

Significance of the effect:

1 the drug has a significant effect

2 the drug has no significant effect

3 the drug has either a significant or non significant effect (depending on the outcome or the exposition)

**CONCLUSION**

In case of subgroups analysis, results vary across subgroups _1_ Yes _0_ No _2_ NR _3_ NA

If yes, it changes the degree of significativity (from significant results to non significant, or vice versa) _1_ Yes _0_ No _2_ NR

If yes, it changes the level of risk (from an excess of risk to a preventive risk, or vice versa) _1_ Yes _0_ No _2_ NR

In case of sensitivity analyses, results vary across sensitivity analyses _1_ Yes _0_ No _2_ NR _3_ NA

If yes, it changes the degree of significativity (from significant results to non significant, or vice versa) _1_ Yes _0_ No _2_ NR

If yes, it changes the level of risk (from an excess of risk to a preventive risk, or vice versa) _1_ Yes _0_ No _2_ NR

In case of comparison of different designs, results vary across designs _1_ Yes _0_ No _2_ NR _3_ NA

If yes, it changes the degree of significativity (from significant results to non significant, or vice versa) _1_ Yes _0_ No _2_ NR

If yes, it changes the level of risk (from an excess of risk to a preventive risk, or vice versa) _1_ Yes _0_ No _2_ NR

Minor validity assumptions of self-controlled designs

|  | Yes | No |
| --- | --- | --- |
| **For CCO** |  |  |
| the opportunity of event is the same during case and control time periods |  |  |
| no time trend in exposure |  |  |
| If time trend in exposure, CTC use |  |  |
| **For SCCS** |  |  |
| Independence between two consecutive events when recurrent |  |  |
| If no independence between two consecutive events, use of adapted design |  |  |
| Probability of further exposure not affected by previous events |  |  |
| If probability of exposure affected by previous event, use of adapted design |  |  |
| Event do not affect the short term mortality probability |  |  |
| If short term mortality affected by the event, use of adapted design |  |  |

# Supplementary Material S3: Articles included in the review

1. Chang CH, Chen HC, Lin JW, Kuo CW, Shau WY, Lai MS. Risk of hospitalization for upper gastrointestinal adverse events associated with nonsteroidal anti-inflammatory drugs: a nationwide case-crossover study in Taiwan. Pharmacoepidemiol Drug Saf. 2011 Jul; 20(7):763-71. DOI:10.1002/pds.2140
2. Chang CH, Lin JW, Chen HC, Kuo CW, Shau WY, Lai MS. Non-steroidal anti-inflammatory drugs and risk of lower gastrointestinal adverse events: a nationwide study in Taiwan. Gut. 2011 Oct; 60(10):1372-8. DOI:10.1136/gut.2010.229906
3. Choi NK, Chang Y, Jung SY, Choi YK, Lee J, Lee JH, et al. A population-based case-crossover study of polyethylene glycol use and acute renal failure risk in the elderly. World J Gastroenterol. 2011 Feb 7; 17(5):651-6. DOI:10.3748/wjg.v17.i5.651
4. Douglas IJ, Evans SJ, Hingorani AD, Grosso AM, Timmis A, Hemingway H, et al. Clopidogrel and interaction with proton pump inhibitors: comparison between cohort and within person study designs. BMJ. 2012; 345:e4388. DOI:10.1136/bmj.e4388
5. Gribbin J, Hubbard R, Gladman J, Smith C, Lewis S. Serotonin-norepinephrine reuptake inhibitor antidepressants and the risk of falls in older people: case-control and case-series analysis of a large UK primary care database. Drugs Aging. 2011 Nov 1; 28(11):895-902. DOI:10.2165/11592860-000000000-00000
6. Gribbin J, Hubbard R, Gladman J, Smith C, Lewis S. Risk of falls associated with antihypertensive medication: self-controlled case series. Pharmacoepidemiol Drug Saf. 2011 Aug; 20(8):879-84. DOI:10.1002/pds.2176
7. Lee CH, Wang JD, Chen PC. Risk of liver injury associated with Chinese herbal products containing radix bupleuri in 639,779 patients with hepatitis B virus infection. PLoS One. 2011; 6(1):e16064. DOI:10.1371/journal.pone.0016064
8. Lee YC, Chang CH, Lin JW, Chen HC, Lin MS, Lai MS. Non-steroidal anti-inflammatory drugs use and risk of upper gastrointestinal adverse events in cirrhotic patients. Liver Int. 2012 May; 32(5):859-66. DOI:10.1111/j.1478-3231.2011.02739.x
9. Risselada R, Straatman H, van Kooten F, Dippel DW, van der Lugt A, Niessen WJ, et al. Platelet aggregation inhibitors, vitamin K antagonists and risk of subarachnoid hemorrhage. J Thromb Haemost. 2011 Mar; 9(3):517-23. DOI:10.1111/j.1538-7836.2010.04170.x
10. Rogers MA, Levine DA, Blumberg N, Flanders SA, Chopra V, Langa KM. Triggers of hospitalization for venous thromboembolism. Circulation. 2012 May 1; 125(17):2092-9. DOI:10.1161/CIRCULATIONAHA.111.084467
11. Seitz DP, Campbell RJ, Bell CM, Gill SS, Gruneir A, Herrmann N, et al. Short-term exposure to antidepressant drugs and risk of acute angle-closure glaucoma among older adults. J Clin Psychopharmacol. 2012 Jun; 32(3):403-7. DOI:10.1097/JCP.0b013e31825420a1
12. Shau WY, Chen HC, Chen ST, Chou HW, Chang CH, Kuo CW, et al. Risk of new acute myocardial infarction hospitalization associated with use of oral and parenteral non-steroidal anti-inflammation drugs (NSAIDs): a case-crossover study of Taiwan's National Health Insurance claims database and review of current evidence. BMC Cardiovasc Disord. 2012; 12:4. DOI:10.1186/1471-2261-12-4
13. Smith SW, Sato M, Gore SD, Baer MR, Ke X, McNally D, et al. Erythropoiesis-stimulating agents are not associated with increased risk of thrombosis in patients with myelodysplastic syndromes. Haematologica. 2012 Jan; 97(1):15-20. DOI:10.3324/haematol.2011.051755
14. Stowe J, Andrews N, Bryan P, Seabroke S, Miller E. Risk of convulsions in children after monovalent H1N1 (2009) and trivalent influenza vaccines: a database study. Vaccine. 2011 Nov 28; 29(51):9467-72. DOI:10.1016/j.vaccine.2011.10.029
15. Wilson K, Hawken S, Kwong JC, Deeks S, Crowcroft NS, Van Walraven C, et al. Adverse events following 12 and 18 month vaccinations: a population-based, self-controlled case series analysis. PLoS One. 2011; 6(12):e27897. DOI:10.1371/journal.pone.0027897
16. Wise BL, Peloquin C, Choi H, Lane NE, Zhang Y. Impact of age, sex, obesity, and steroid use on quinolone-associated tendon disorders. Am J Med. 2012 Dec; 125(12):1228 e23- e28. DOI:10.1016/j.amjmed.2012.05.027
17. Wu CS, Wang SC, Cheng YC, Gau SS. Association of cerebrovascular events with antidepressant use: a case-crossover study. Am J Psychiatry. 2011 May; 168(5):511-21. DOI:10.1176/appi.ajp.2010.10071064
18. Yang YH, Lai JN, Lee CH, Wang JD, Chen PC. Increased risk of hospitalization related to motor vehicle accidents among people taking zolpidem: a case-crossover study. J Epidemiol. 2011; 21(1):37-43
19. Benchimol EI, Hawken S, Kwong JC, Wilson K. Safety and utilization of influenza immunization in children with inflammatory bowel disease. Pediatrics. 2013 Jun; 131(6):e1811-20. DOI:10.1542/peds.2012-3567
20. Berry SD, Lee Y, Cai S, Dore DD. Nonbenzodiazepine sleep medication use and hip fractures in nursing home residents. JAMA Intern Med. 2013 May 13; 173(9):754-61. DOI:10.1001/jamainternmed.2013.3795
21. Bird ST, Delaney JA, Brophy JM, Etminan M, Skeldon SC, Hartzema AG. Tamsulosin treatment for benign prostatic hyperplasia and risk of severe hypotension in men aged 40-85 years in the United States: risk window analyses using between and within patient methodology. BMJ. 2013; 347:f6320. DOI:10.1136/bmj.f6320
22. Bird ST, Etminan M, Brophy JM, Hartzema AG, Delaney JA. Risk of acute kidney injury associated with the use of fluoroquinolones. CMAJ. 2013 Jul 9; 185(10):E475-82. DOI:10.1503/cmaj.121730
23. Butt DA, Mamdani M, Austin PC, Tu K, Gomes T, Glazier RH. The risk of hip fracture after initiating antihypertensive drugs in the elderly. Arch Intern Med. 2012 Dec 10; 172(22):1739-44. DOI:10.1001/2013.jamainternmed.469
24. Butt DA, Mamdani M, Austin PC, Tu K, Gomes T, Glazier RH. The risk of falls on initiation of antihypertensive drugs in the elderly. Osteoporos Int. 2013 Oct; 24(10):2649-57. DOI:10.1007/s00198-013-2369-7
25. Choi NK, Lee J, Chang Y, Kim YJ, Kim JY, Song HJ, et al. Acute renal failure following oral sodium phosphate bowel preparation: a nationwide case-crossover study. Endoscopy. 2014 Jun; 46(6):465-70. DOI:10.1055/s-0034-1365419
26. Chui CS, Man KK, Cheng CL, Chan EW, Lau WC, Cheng VC, et al. An investigation of the potential association between retinal detachment and oral fluoroquinolones: a self-controlled case series study. J Antimicrob Chemother. 2014 Sep; 69(9):2563-7. DOI:10.1093/jac/dku145
27. Dodd CN, Romio SA, Black S, Vellozzi C, Andrews N, Sturkenboom M, et al. International collaboration to assess the risk of Guillain Barre Syndrome following Influenza A (H1N1) 2009 monovalent vaccines. Vaccine. 2013 Sep 13; 31(40):4448-58. DOI:10.1016/j.vaccine.2013.06.032
28. Fardet L, Nazareth I, Whitaker HJ, Petersen I. Severe neuropsychiatric outcomes following discontinuation of long-term glucocorticoid therapy: a cohort study. J Clin Psychiatry. 2013 Apr; 74(4):e281-6. DOI:10.4088/JCP.12m08034
29. Li X, Zhang Z, Duke J. Glucagon-like peptide 1-based therapies and risk of pancreatitis: a self-controlled case series analysis. Pharmacoepidemiol Drug Saf. 2014 Mar; 23(3):234-9
30. Liu CL, Shau WY, Chang CH, Wu CS, Lai MS. Pneumonia risk and use of angiotensin-converting enzyme inhibitors and angiotensin II receptor blockers. J Epidemiol. 2013 Sep 5; 23(5):344-50
31. Liu CL, Shau WY, Wu CS, Lai MS. Angiotensin-converting enzyme inhibitor/angiotensin II receptor blockers and pneumonia risk among stroke patients. J Hypertens. 2012 Nov; 30(11):2223-9. DOI:10.1097/HJH.0b013e328357a87a
32. Orriols L, Wilchesky M, Lagarde E, Suissa S. Prescription of antidepressants and the risk of road traffic crash in the elderly: a case-crossover study. Br J Clin Pharmacol. 2013 Nov; 76(5):810-5. DOI:10.1111/bcp.12090
33. Raman SR, Marshall SW, Haynes K, Gaynes BN, Naftel AJ, Sturmer T. Stimulant treatment and injury among children with attention deficit hyperactivity disorder: an application of the self-controlled case series study design. Inj Prev. 2013 Jun; 19(3):164-70. DOI:10.1136/injuryprev-2012-040483
34. Wang YP, Chen YT, Tsai CF, Li SY, Luo JC, Wang SJ, et al. Short-term use of serotonin reuptake inhibitors and risk of upper gastrointestinal bleeding. Am J Psychiatry. 2014 Jan; 171(1):54-61. DOI:10.1176/appi.ajp.2013.12111467
35. Wu CS, Wang SC, Gau SS, Tsai HJ, Cheng YC. Association of stroke with the receptor-binding profiles of antipsychotics-a case-crossover study. Biol Psychiatry. 2013 Mar 1; 73(5):414-21. DOI:10.1016/j.biopsych.2012.07.006
36. Wu IC, Lin MY, Yu FJ, Hsieh HM, Chiu KF, Wu MT. A short-term effect of low-dose aspirin on major hemorrhagic risks in primary prevention: a case-crossover design. PLoS One. 2014; 9(5):e98326. DOI:10.1371/journal.pone.0098326
37. Abrams JY, Weintraub ES, Baggs JM, McCarthy NL, Schonberger LB, Lee GM, et al. Childhood vaccines and Kawasaki disease, Vaccine Safety Datalink, 1996-2006. Vaccine. 2015 Jan 3; 33(2):382-7. DOI:10.1016/j.vaccine.2014.10.044
38. Andrews N, Stowe J, Miller E, Svanstrom H, Johansen K, Bonhoeffer J, et al. A collaborative approach to investigating the risk of thrombocytopenic purpura after measles-mumps-rubella vaccination in England and Denmark. Vaccine. 2012 Apr 19; 30(19):3042-6. DOI:10.1016/j.vaccine.2011.06.009
39. Arnheim-Dahlstrom L, Hallgren J, Weibull CE, Sparen P. Risk of presentation to hospital with epileptic seizures after vaccination with monovalent AS03 adjuvanted pandemic A/H1N1 2009 influenza vaccine (Pandemrix): self controlled case series study. BMJ. 2012; 345:e7594. DOI:10.1136/bmj.e7594
40. Berard A, Azoulay L, Nakhai-Pour HR, Moussally K. Isotretinoin and the risk of cardiovascular, cerebrovascular and thromboembolic disorders. Dermatology. 2011; 223(1):45-51. DOI:10.1159/000330326
41. Berry SD, Zhu Y, Choi H, Kiel DP, Zhang Y. Diuretic initiation and the acute risk of hip fracture. Osteoporos Int. 2013 Feb; 24(2):689-95. DOI:10.1007/s00198-012-2053-3
42. Bjorkenstam C, Moller J, Ringback G, Salmi P, Hallqvist J, Ljung R. An association between initiation of selective serotonin reuptake inhibitors and suicide - a nationwide register-based case-crossover study. PLoS One. 2013; 8(9):e73973. DOI:10.1371/journal.pone.0073973
43. Brauer R, Smeeth L, Anaya-Izquierdo K, Timmis A, Denaxas SC, Farrington CP, et al. Antipsychotic drugs and risks of myocardial infarction: a self-controlled case series study. Eur Heart J. 2015 Apr 21; 36(16):984-92. DOI:10.1093/eurheartj/ehu263
44. Carlin JB, Macartney KK, Lee KJ, Quinn HE, Buttery J, Lopert R, et al. Intussusception risk and disease prevention associated with rotavirus vaccines in Australia's National Immunization Program. Clin Infect Dis. 2013 Nov; 57(10):1427-34. DOI:10.1093/cid/cit520
45. Chang CH, Lin JW, Lin CH, Chen HC, Hwang JJ, Lai MS. Effectiveness and safety of extracranial carotid stent placement: a nationwide self-controlled case-series study. J Formos Med Assoc. 2015 Mar; 114(3):274-81. DOI:10.1016/j.jfma.2014.05.001
46. Choi NK, Lee J, Chang Y, Jung SY, Kim YJ, Lee SM, et al. Polyethylene glycol bowel preparation does not eliminate the risk of acute renal failure: a population-based case-crossover study. Endoscopy. 2013; 45(3):208-13. DOI:10.1055/s-0032-1326031
47. Dassanayake TL, Jones AL, Michie PT, Carter GL, McElduff P, Stokes BJ, et al. Risk of road traffic accidents in patients discharged following treatment for psychotropic drug overdose: a self-controlled case series study in Australia. CNS Drugs. 2012 Mar 1; 26(3):269-76. DOI:10.2165/11599790-000000000-00000
48. De Wals P, Deceuninck G, Toth E, Boulianne N, Brunet D, Boucher RM, et al. Risk of Guillain-Barre syndrome following H1N1 influenza vaccination in Quebec. JAMA. 2012 Jul 11; 308(2):175-81. DOI:10.1001/jama.2012.7342
49. Donegan K, Beau-Lejdstrom R, King B, Seabroke S, Thomson A, Bryan P. Bivalent human papillomavirus vaccine and the risk of fatigue syndromes in girls in the UK. Vaccine. 2013 Oct 9; 31(43):4961-7. DOI:10.1016/j.vaccine.2013.08.024
50. Douglas IJ, Langham J, Bhaskaran K, Brauer R, Smeeth L. Orlistat and the risk of acute liver injury: self controlled case series study in UK Clinical Practice Research Datalink. BMJ. 2013; 346:f1936. DOI:10.1136/bmj.f1936
51. Fife D, Zhu V, Voss E, Levy-Clarke G, Ryan P. Exposure to oral fluoroquinolones and the risk of retinal detachment: retrospective analyses of two large healthcare databases. Drug Saf. 2014 Mar; 37(3):171-82. DOI:10.1007/s40264-014-0138-y
52. Fosbol EL, Olsen AM, Olesen JB, Andersson C, Kober L, Torp-Pedersen C, et al. Use of nonsteroidal anti-inflammatory drugs among healthy people and specific cerebrovascular safety. Int J Stroke. 2014 Oct; 9(7):943-5. DOI:10.1111/j.1747-4949.2012.00863.x
53. Gwini SM, Coupland CA, Siriwardena AN. The effect of influenza vaccination on risk of acute myocardial infarction: self-controlled case-series study. Vaccine. 2011 Feb 1; 29(6):1145-9. DOI:10.1016/j.vaccine.2010.12.017
54. Hambidge SJ, Newcomer SR, Narwaney KJ, Glanz JM, Daley MF, Xu S, et al. Timely versus delayed early childhood vaccination and seizures. Pediatrics. 2014 Jun; 133(6):e1492-9. DOI:10.1542/peds.2013-3429
55. Hambidge SJ, Ross C, Glanz J, McClure D, Daley MF, Xu S, et al. Trivalent inactivated influenza vaccine is not associated with sickle cell crises in children. Pediatrics. 2012 Jan; 129(1):e54-9. DOI:10.1542/peds.2011-1294
56. Hambidge SJ, Ross C, McClure D, Glanz J. Trivalent inactivated influenza vaccine is not associated with sickle cell hospitalizations in adults from a large cohort. Vaccine. 2011 Oct 26; 29(46):8179-81. DOI:10.1016/j.vaccine.2011.09.002
57. Hanf M, Quantin C, Farrington P, Benzenine E, Hocine NM, Velten M, et al. Validation of the French national health insurance information system as a tool in vaccine safety assessment: application to febrile convulsions after pediatric measles/mumps/rubella immunization. Vaccine. 2013 Dec 2; 31(49):5856-62. DOI:10.1016/j.vaccine.2013.09.052
58. Hawken S, Manuel DG, Deeks SL, Kwong JC, Crowcroft NS, Wilson K. Underestimating the safety benefits of a new vaccine: the impact of acellular pertussis vaccine versus whole-cell pertussis vaccine on health services utilization. Am J Epidemiol. 2012 Dec 1; 176(11):1035-42. DOI:10.1093/aje/kws167
59. Huang WT, Tang FW, Yang SE, Chih YC, Chuang JH. Safety of inactivated monovalent pandemic (H1N1) 2009 vaccination during pregnancy: a population-based study in Taiwan. Vaccine. 2014 Nov 12; 32(48):6463-8. DOI:10.1016/j.vaccine.2014.09.054
60. Huang WT, Yang HW, Liao TL, Wu WJ, Yang SE, Chih YC, et al. Safety of pandemic (H1N1) 2009 monovalent vaccines in taiwan: a self-controlled case series study. PLoS One. 2013; 8(3):e58827. DOI:10.1371/journal.pone.0058827
61. Jung SY, Choi NK, Kim JY, Chang Y, Song HJ, Lee J, et al. Short-acting nifedipine and risk of stroke in elderly hypertensive patients. Neurology. 2011 Sep 27; 77(13):1229-34. DOI:10.1212/WNL.0b013e318230201a
62. Kang DY, Park S, Rhee CW, Kim YJ, Choi NK, Lee J, et al. Zolpidem use and risk of fracture in elderly insomnia patients. J Prev Med Public Health. 2012 Jul; 45(4):219-26. DOI:10.3961/jpmph.2012.45.4.219
63. Koopmans PC, Bos JH, de Jong van den Berg LT. Are antibiotics related to oral combination contraceptive failures in the Netherlands? A case-crossover study. Pharmacoepidemiol Drug Saf. 2012 Aug; 21(8):865-71. DOI:10.1002/pds.3267
64. Lee CH, Jang EJ, Hyun MK, Lee NR, Kim K, Yim JJ. Risk of hospital admission or emergency room visit for pneumonia in patients using respiratory inhalers: a case-crossover study. Respirology. 2013 Oct; 18(7):1116-27. DOI:10.1111/resp.12127
65. Lee CH, Wang JD, Chen PC. Case-crossover design: an alternative strategy for detecting drug-induced liver injury. J Clin Epidemiol. 2012 May; 65(5):560-7. DOI:10.1016/j.jclinepi.2011.11.002
66. Lee JK, Lee J, Park SS, Heo EY, Park YS, Lee CH, et al. Effect of inhalers on the development of haemoptysis in patients with non-cystic fibrosis bronchiectasis. Int J Tuberc Lung Dis. 2014 Mar; 18(3):363-70. DOI:10.5588/ijtld.13.0255
67. Lin ST, Chen CC, Tsang HY, Lee CS, Yang P, Cheng KD, et al. Association between antipsychotic use and risk of acute myocardial infarction: a nationwide case-crossover study. Circulation. 2014 Jul 15; 130(3):235-43. DOI:10.1161/circulationaha.114.008779
68. Macartney KK, Gidding HF, Trinh L, Wang H, McRae J, Crawford N, et al. Febrile seizures following measles and varicella vaccines in young children in Australia. Vaccine. 2015 Mar 10; 33(11):1412-7. DOI:10.1016/j.vaccine.2014.10.071
69. Man KK, Chan EW, Coghill D, Douglas I, Ip P, Leung LP, et al. Methylphenidate and the risk of trauma. Pediatrics. 2015 Jan; 135(1):40-8. DOI:10.1542/peds.2014-1738
70. Masclee GM, Valkhoff VE, Coloma PM, de Ridder M, Romio S, Schuemie MJ, et al. Risk of upper gastrointestinal bleeding from different drug combinations. Gastroenterology. 2014 Oct; 147(4):784-92 e9; quiz e13-4. DOI:10.1053/j.gastro.2014.06.007
71. Meuleners LB, Duke J, Lee AH, Palamara P, Hildebrand J, Ng JQ. Psychoactive medications and crash involvement requiring hospitalization for older drivers: a population-based study. J Am Geriatr Soc. 2011 Sep; 59(9):1575-80. DOI:10.1111/j.1532-5415.2011.03561.x
72. Mullooly JP, Schuler R, Mesa J, Drew L, DeStefano F. Wheezing lower respiratory disease and vaccination of premature infants. Vaccine. 2011 Oct 13; 29(44):7611-7. DOI:10.1016/j.vaccine.2011.08.022
73. Orriols L, Foubert-Samier A, Gadegbeku B, Delorme B, Tricotel A, Philip P, et al. Prescription of antiepileptics and the risk of road traffic crash. J Clin Pharmacol. 2013 Mar; 53(3):339-44. DOI:10.1002/jcph.10
74. Pariente A, Fourrier-Reglat A, Ducruet T, Farrington P, Beland SG, Dartigues JF, et al. Antipsychotic use and myocardial infarction in older patients with treated dementia. Arch Intern Med. 2012 Apr 23; 172(8):648-53; discussion 54-5. DOI:10.1001/archinternmed.2012.28
75. Pottegard A, dePont Christensen R, Wang SV, Gagne JJ, Larsen TB, Hallas J. Pharmacoepidemiological assessment of drug interactions with vitamin K antagonists. Pharmacoepidemiol Drug Saf. 2014 Nov; 23(11):1160-7. DOI:10.1002/pds.3714
76. Pratt N, Roughead EE, Ramsay E, Salter A, Ryan P. Risk of hospitalization for hip fracture and pneumonia associated with antipsychotic prescribing in the elderly: a self-controlled case-series analysis in an Australian health care claims database. Drug Saf. 2011 Jul 1; 34(7):567-75. DOI:10.2165/11588470-000000000-00000
77. Pratt NL, Ramsay EN, Kemp A, Kalisch-Ellett LM, Shakib S, Caughey GE, et al. Ranibizumab and risk of hospitalisation for ischaemic stroke and myocardial infarction in patients with age-related macular degeneration: a self-controlled case-series analysis. Drug Saf. 2014 Dec; 37(12):1021-7. DOI:10.1007/s40264-014-0231-2
78. Price SD, Holman CD, Sanfilippo FM, Emery JD. Use of case-time-control design in pharmacovigilance applications: exploration with high-risk medications and unplanned hospital admissions in the Western Australian elderly. Pharmacoepidemiol Drug Saf. 2013 Nov; 22(11):1159-70. DOI:10.1002/pds.3469
79. Price SD, Holman CD, Sanfilippo FM, Emery JD. Impact of specific Beers Criteria medications on associations between drug exposure and unplanned hospitalisation in elderly patients taking high-risk drugs: a case-time-control study in Western Australia. Drugs Aging. 2014 Apr; 31(4):311-25. DOI:10.1007/s40266-014-0164-6
80. Price SD, Holman CD, Sanfilippo FM, Emery JD. Association between potentially inappropriate medications from the Beers criteria and the risk of unplanned hospitalization in elderly patients. Ann Pharmacother. 2014 Jan; 48(1):6-16. DOI:10.1177/1060028013504904
81. Price SD, Holman CD, Sanfilippo FM, Emery JD. Are high-care nursing home residents at greater risk of unplanned hospital admission than other elderly patients when exposed to Beers potentially inappropriate medications? Geriatr Gerontol Int. 2014 Oct; 14(4):934-41. DOI:10.1111/ggi.12200
82. Price SD, Holman CD, Sanfilippo FM, Emery JD. Does ongoing general practitioner care in elderly patients help reduce the risk of unplanned hospitalization related to Beers potentially inappropriate medications? Geriatr Gerontol Int. 2015 Aug; 15(8):1031-9. DOI:10.1111/ggi.12400
83. Quinn HE, Wood NJ, Cannings KL, Dey A, Wang H, Menzies RI, et al. Intussusception after monovalent human rotavirus vaccine in Australia: severity and comparison of using healthcare database records versus case confirmation to assess risk. Pediatr Infect Dis J. 2014 Sep; 33(9):959-65. DOI:10.1097/inf.0000000000000362
84. Ramsay EN, Pratt NL, Ryan P, Roughead EE. Proton pump inhibitors and the risk of pneumonia: a comparison of cohort and self-controlled case series designs. BMC Med Res Methodol. 2013; 13:82. DOI:10.1186/1471-2288-13-82
85. Ramsay EN, Roughead EE, Ewald B, Pratt NL, Ryan P. A self-controlled case series to assess the effectiveness of beta blockers for heart failure in reducing hospitalisations in the elderly. BMC Med Res Methodol. 2011; 11:106. DOI:10.1186/1471-2288-11-106
86. Ravera S, van Rein N, de Gier JJ, de Jong-van den Berg LT. A comparison of pharmacoepidemiological study designs in medication use and traffic safety research. Eur J Epidemiol. 2012 Jun; 27(6):473-81. DOI:10.1007/s10654-012-9689-3
87. Rogers MA, Micic D, Blumberg N, Young VB, Aronoff DM. Storage duration of red blood cell transfusion and Clostridium difficile infection: a within person comparison. PLoS One. 2014; 9(2):e89332. DOI:10.1371/journal.pone.0089332
88. Romio S, Weibel D, Dieleman JP, Olberg HK, de Vries CS, Sammon C, et al. Guillain-Barre syndrome and adjuvanted pandemic influenza A (H1N1) 2009 vaccines: a multinational self-controlled case series in Europe. PLoS One. 2014; 9(1):e82222. DOI:10.1371/journal.pone.0082222
89. Schurink-Van't Klooster TM, de Ridder MA, Kemmeren JM, van der Lei J, Dekker F, Sturkenboom M, et al. Examining a possible association between human papilloma virus (HPV) vaccination and migraine: results of a cohort study in the Netherlands. Eur J Pediatr. 2015 May; 174(5):641-9. DOI:10.1007/s00431-014-2444-x
90. Shin JY, Choi NK, Jung SY, Lee J, Kwon JS, Park BJ. Risk of ischemic stroke with the use of risperidone, quetiapine and olanzapine in elderly patients: a population-based, case-crossover study. J Psychopharmacol. 2013 Jul; 27(7):638-44. DOI:10.1177/0269881113482530
91. Soderberg KC, Laflamme L, Moller J. Newly initiated opioid treatment and the risk of fall-related injuries. A nationwide, register-based, case-crossover study in Sweden. CNS Drugs. 2013 Feb; 27(2):155-61. DOI:10.1007/s40263-013-0038-1
92. Sun Y, Christensen J, Hviid A, Li J, Vedsted P, Olsen J, et al. Risk of febrile seizures and epilepsy after vaccination with diphtheria, tetanus, acellular pertussis, inactivated poliovirus, and Haemophilus influenzae type B. JAMA. 2012 Feb 22; 307(8):823-31. DOI:10.1001/jama.2012.165
93. Sun Y, Wu CS, Olsen J. Trimethoprim use before pregnancy and risk of congenital malformation: reanalyzed using a case-crossover design and a case-time-control design. Pharmacoepidemiol Drug Saf. 2014 Oct; 23(10):1076-83. DOI:10.1002/pds.3691
94. Toh S, Mitchell AA, Anderka M, de Jong-van den Berg LT, Hernandez-Diaz S. Antibiotics and oral contraceptive failure - a case-crossover study. Contraception. 2011 May; 83(5):418-25. DOI:10.1016/j.contraception.2010.08.020
95. Tseng HF, Liu A, Sy L, Marcy SM, Fireman B, Weintraub E, et al. Safety of zoster vaccine in adults from a large managed-care cohort: a Vaccine Safety Datalink study. J Intern Med. 2012 May; 271(5):510-20. DOI:10.1111/j.1365-2796.2011.02474.x
96. Tseng HF, Sy LS, Qian L, Marcy SM, Jackson LA, Glanz J, et al. Safety of a tetanus-diphtheria-acellular pertussis vaccine when used off-label in an elderly population. Clin Infect Dis. 2013 Feb; 56(3):315-21. DOI:10.1093/cid/cis871
97. Turkiewicz A, Vicente RP, Ohlsson H, Tyden P, Merlo J. Revising the link between proton-pump inhibitors and risk of acute myocardial infarction-a case-crossover analysis. Eur J Clin Pharmacol. 2015 Jan; 71(1):125-9. DOI:10.1007/s00228-014-1779-6
98. Ueyama H, Hinotsu S, Tanaka S, Urushihara H, Nakamura M, Nakamura Y, et al. Application of a self-controlled case series study to a database study in children. Drug Saf. 2014 Apr; 37(4):259-68. DOI:10.1007/s40264-014-0148-9
99. Wang S, Linkletter C, Dore D, Mor V, Buka S, Maclure M. Age, antipsychotics, and the risk of ischemic stroke in the Veterans Health Administration. Stroke. 2012 Jan; 43(1):28-31. DOI:10.1161/strokeaha.111.617191
100. Weeke P, Jensen A, Folke F, Gislason GH, Olesen JB, Andersson C, et al. Antidepressant use and risk of out-of-hospital cardiac arrest: a nationwide case-time-control study. Clin Pharmacol Ther. 2012 Jul; 92(1):72-9. DOI:10.1038/clpt.2011.368
101. Weeke P, Jensen A, Folke F, Gislason GH, Olesen JB, Fosbol EL, et al. Antipsychotics and associated risk of out-of-hospital cardiac arrest. Clin Pharmacol Ther. 2014 Oct; 96(4):490-7. DOI:10.1038/clpt.2014.139
102. Wijlaars LP, Nazareth I, Whitaker HJ, Evans SJ, Petersen I. Suicide-related events in young people following prescription of SSRIs and other antidepressants: a self-controlled case series analysis. BMJ Open. 2013; 3(9):e003247. DOI:10.1136/bmjopen-2013-003247
103. Wilson K, Ducharme R, Ward B, Hawken S. Increased emergency room visits or hospital admissions in females after 12-month MMR vaccination, but no difference after vaccinations given at a younger age. Vaccine. 2014 Feb 26; 32(10):1153-9. DOI:10.1016/j.vaccine.2014.01.010
104. Wilson K, Hawken S. Incidence of adverse events in premature children following 2-month vaccination. Hum Vaccin Immunother. 2012 May; 8(5):592-5. DOI:10.4161/hv.19377
105. Wilson K, Hawken S, Kwong JC, Deeks SL, Manuel DG, Henningsen KH, et al. Impact of birth weight at term on rates of emergency room visits and hospital admissions following vaccination at 2 months of age. Vaccine. 2011 Oct 26; 29(46):8267-74. DOI:10.1016/j.vaccine.2011.08.107
106. Wilson K, Hawken S, Potter BK, Chakraborty P, Kwong J, Crowcroft N, et al. Patterns of emergency room visits, admissions and death following recommended pediatric vaccinations - a population based study of 969,519 vaccination events. Vaccine. 2011 May 12; 29(21):3746-52. DOI:10.1016/j.vaccine.2011.03.044
107. Wright AJ, Gomes T, Mamdani MM, Horn JR, Juurlink DN. The risk of hypotension following co-prescription of macrolide antibiotics and calcium-channel blockers. CMAJ. 2011 Feb 22; 183(3):303-7. DOI:10.1503/cmaj.100702

# Supplementary MaterialS4a: Validity assumptions for each article describing a case-crossover design

| **Reference (details in Appendix 3)** | **Type of exposure** | **Intermittent exposure** | **Outcome of interest** | **Rare event** | **Abrupt onset event** | **Same opportunity of exposure during case and control periods** | **Absence of time trend in exposure, or design adaptation, such as use of case-time-control design** |
| --- | --- | --- | --- | --- | --- | --- | --- |
| Berry, JAMA Intern Med, 2013 | Non-benzodiazepine hypnotic drugs | unclear | Hip fracture | Y | Y | Y | N |
| Bird, CMAJ, 2013 | Fluoroquinolones | Y | Acute kidney injury | Y | Y | Y | Y |
| Choi, Endoscopy, 2014 | Oral sodium phosphate | Y | Acute renal failure | Y | Y | Y | Y |
| Liu, J Epidemiol 2014 | ACE inhibitors | N | Pneumonia | Y | Y | Y | Y |
| Orriols, BJCP 2013 | Antidepressant | Y | Car crash | Y | Y | N | Y |
| Wu, Biol Psychiatry 2013 | Psychotropes | N | Stroke | Y | Y | Y | N |
| Wu, Plos One, 2014 | Low dose aspirin | N | Hemorragia (cerebral or gastrointestinal) | Y | Y | Y | Y |
| Wang, Am J Psychiatry, 2014 | Short term use of SSRI antidepressants | Y | UGIB | Y | Y | Y | Y |
| Weiss Smith, Hematologia 2012 | EPO | Y | DVT/PE | Y | Y | Y | Y |
| Chang, J Med Econ, 2011 | NSAID | Y | Hospitalisation for UGIB | Y | Y | Y | Y |
| Chang, Gut, 2011 | NSAID | Y | Hospitalisation for LGIB | Y | Y | Y | Y |
| Choi, World J Gastroenterol, 2011 | PEG | Y | Hospitalisation for acute renal failure | Y | Y | Y | Y |
| Yang, J Epidemiol, 2011 | Benzodiazepine | Y | Car crash | Y | Y | Y | Y |
| Lee, Plos One, 2011 | Radix bupleuri | unclear | Hospitalisation for liver injuries | Y | Y | Y | Y |
| Risselada, J Thrombosis and Haemostasis, 2011 | Anti-platelet agregant Or AVK | N | Hospitalisation for subarachnoidal hemorrhage | Y | Y | Y | Y |
| Wu, Am J Psychiatry 2011 | Antidepressant | N | Hospitalisation for CV events | Y | Y | Y | Y |
| Rogers, Circulation, 2012 | EPO, chemo or antidepressive drug | Y | Hospitalisation for DVT/PE | Y | Y | Y | Y |
| Shau, BMC Cardiovascular Disorders, 2012 | NSAID | Y | New hospitalisation for AMI | Y | Y | Y | Y |
| Seitz, J Clin Psychopharmacol, 2012 | Antidepressant | Y | Acute glaucoma | Y | Y | Y | Y |
| Wise, Am J Med, 2012 | Quinolone | Y | Achilles tendonitis or tendon rupture | Y | Y | Y | N |
| Lee, Liver Int, 2012 | NSAID | Y | UGIB | Y | Y | Y | Y |
| Liu, J Hypertension, 2012 | ACE inhibitors and angiotensin II receptor blockers | N | Hospitalisation for pneumonia | N | Y | Y | Y |
| Berard, Dermatol, 2011 | Isotretinoid prescription | Y | Diagnosis or hospitalizations for MI, stroke, PE, thrombosis, thrombophlebitis | N | Y | Y | Y |
| Lee, In J Tuberc Lung Dis, 2014 | Inhalers | N | Haemoptysis | Y | Y | Y | Y |
| Abrams, Vaccine, 2014 | Vaccines | Y | Kawasaki disease | Y | Y | Y | Y |
| Berry, Osteop Int, 2013 | Diuretic initiation | Y | Hip fracture | Y | Y | Y | Y |
| Björkenstam, Plos One 2013 | Initiation with selective serotonin reuptake inhibitors (SSRI) | Y | Suicide | Y | Y | Y | Y |
| Choi, Endoscopy, 2013 | PEG | Y | Acute renal failure | Y | Y | Y | Y |
| Fosbol, IGS 2014 | NSAID | Y | Ischemic stroke or hemorragic stroke (fatal or non fatal) | Y | Y | Y | Y |
| Jung, Neurol, 2011 | Episodic use of short acting nifedipine | Y | Stroke | Y | Y | Y | N |
| Kang, JPMPH, 2012 | Zolpidem | Y | Fracture | Y | Y | Y | Y |
| Koopmans, PDS, 2012 | Antibiotic use | Y | Breakthrough pregnancies | Y | Y | Y | Y |
| Lee, Respirology 2013 | Inhalers | N | Hospital admission or ED visit for pneumonia | Y | Y | Y | Y |
| Lee, JCE, 2012 | Antituberculosis drugs, antibiotics, and NSAID. | Y | Liver injury | Y | Y | Y | Y |
| Lin, Circulation 2014 | Psychotropes | Y | MI | Y | Y | Y | Y |
| Meuleners, JAGS, 2011 | Benzodiazepines, antidepressant, analgesiques opioïdes | unclear | Car crash | Y | Y | Y | Y |
| Orriols, JCP 2013 | Antiepileptic | N | Car crash | Y | Y | Y | Y |
| Price, PDS, 2013 | Anticoagulants, antirheumatic/NSAID, corticosteroid, antihypertension drugs, cardiac rythm regulators, betablockers, serum lipid-lowering agents | unclear | Unplanned hospitalization | N | Y | Y | Y |
| Price, Drug Aging 2014 | Anticoagulants, antirheumatic/NSAID, corticosteroid, antihypertension drugs, cardiac rythm regulators, betablockers, serum lipid-lowering agents | unclear | Unplanned hospitalization | N | Y | Y | Y |
| Price, ann pharmacol, 2014 | Potentially inappropriate medication | unclear | Unplanned hospitalization | N | Y | Y | Y |
| Price, geriatr gerontol, 2014 | Potentially inappropriate medication | unclear | Unplanned hospitalization | N | Y | oui | Y |
| Price, geriatr gerontol 2015 | Potentially inappropriate medication | unclear | Unplanned hospitalization | N | Y | Y | Y |
| Rogers, Plos One, 2014 | Red blood cell transfusion | Y | Hospitalisation for clostridium difficile infection | Y | Y | Y | Y |
| Shin, J Psychopharmacol, 2013 | Risperidone, quetiapine, olanzapine | N | Ischemic stoke | Y | Y | Y | Y |
| Ravera, Eur J Epid, 2012 | Antipsychotic, anxiolytics, hypnotics, antidepressants, | N | Car crash | Y | Y | Y | Y |
| Soderberg, CNS drug, 2013 | Opioid drugs | Y | Fall related injuries | Y | Y | Y | Y |
| Sun, PDS, 2014 | Trimetrhoprim | Y | Congenital malformation | Y | N | N | Y |
| Toh, Contraception, 2011 | Antibiotics | Y | Oral contraceptive failure (breakthrough pregancy) | Y | Y | Y | Y |
| Wright, CMAJ_ 2014 | Macrolides | Y | Hospitalisation for hypotension or shock | Y | Y | Y | Y |
| Weeke,CPT, 2014 | Antipsychotics | N | Out-of-Hospital cardiac arrest | Y | Y | Y | Y |
| Weeke,CPT, 2012 | Antidepressants | N | Out-of-Hospital cardiac arrest | Y | Y | Y | Y |
| Turkiewicz, A. EJCP. 2014 | Proton pump inhibitors | Y | Hospitalisation for AMI | Y | Y | Y | Y |
| Wang, stroke, 2012 | Antipsychotics | Y | Stroke | Y | Y | Y | Y |

ACE: angiotensin-converting enzyme; AMI: acute myocardial infarction; DVT; Deep vein thrombosis; EPO: erythropoietin; LGIB: lower gastro-intestinal bleeding; NR: not reported; NSAID: non selective anti-inflammatory drug; PE: pulmonary embolism; PEG: polyethylene glycol; UGIB: upper gastro-intestinal bleeding

# Supplementary Material S4b: Validity assumptions for each article describing a self-controlled case-series design

| **Reference (details in Appendix 3)** | **Type of exposure** | **Intermittent exposure** | **Outcome of interest** | **Rare or recurrent event** | **Abrupt onset event** | **Independent recurrences or design adaptation, such as considering the 1st event** | **Event-independent exposure or design adaptation, such as excluding person-times before exposure or pseudolikelihood method** | **Event-independant censoring or extension of the case series method, involving the time interval between the event and end of the actual observation period** |
| --- | --- | --- | --- | --- | --- | --- | --- | --- |
| Benchimol, Pediatrics, 2013 | Influenza immunization | Y | Hospitalization, ED visit, physician office visit, IBD related-events | Y | Y | Y | Y | Y |
| Bird, BMJ, 2013 | Alpha blokers | N | Severe hypotension requiring hospitalisation | Y | Y | Y | Y | Y |
| Butt, Osteoporos Int, 2013 | Initiation of antihypertensive drug | Y | Falls | Y | Y | Y | Y | Y |
| Chui, J Antimicrob Chemother 2014 | Fluoroquinolones | Y | Retinal detachment | Y | Y | Y | Y | Y |
| Fardet, J Clin Psychiatry, 2013 | Glucocorticoide discontinuation | Y | Depression, bipolar disorders, délirium, mania, panic attack, suicide or suicide attempt | Y | N | Y | N | N |
| Li, PDS, 2014 | GLP-1-based therapies | N | Acute pancreatitis | Y | Y | Y | Y | Y |
| Raman, Injury Prevention, 2013 | Stimulant | Y | Injury | Y | Y | Y | Y | Y |
| Gribbin, PDS 2011 | Antihypertensive | N | Falls | Y | Y | Y | Y | Y |
| Douglas, BMJ, 2012 | PPI | N | CV events or death | Y | Y | Y | Y | Y |
| Gribbin, Drug Aging, 2012 | Antidepressive drug | Y | Falls | Y | Y | Y | Y | Y |
| Wilson, Plos One, 2011 | MMR or meningo C vaccine | Y | ED visit or hospital admission | Y | Y | Y | Y | Y |
| Stowe, Vaccine, 2011 | H1N1 influnza vaccine | Y | Convulsions | Y | Y | Y | Y | Y |
| Butt, Arch Int Med, 2012 | Antihypertensive therapy initiation | Y | First occurrence for a proximal femoral fracture | Y | Y | Y | Y | Y |
| Dodd, Vaccine, 2013 | Vaccin H1N1 | Y | GBS diagnosis or hospitalisation for GBS | Y | Y | Y | Y | Y |
| Gwini, Vaccine, 2011 | Influenza immunization | Y | First AMI | Y | Y | Y | Y | N |
| Abrams, Vaccine, 2014 | Vaccines | Y | Kawasaki disease | Y | Y | Y | N | Y |
| Andrews, Vaccine, 2012 | MMR vaccine | Y | Thrombocytopenic purpura | Y | Y | Y | Y | Y |
| Arnheim-Dahlström, BMJ, 2012 | H1N1 influnza vaccine | Y | Seizure | Y | Y | Y | Y | Y |
| brauer, eur Heart J, 2014 | Initiation of antipsychotic use | Y | MI | Y | Y | Y | N | Y |
| Carlin, CID 2013 | Rotavirus vaccins | Y | Intussusception | Y | Y | Y | Y | Y |
| Chang, J formosan med ass, 2014 | Carotid angioplasty and stent | N | Ischemic stroke or death | Y | Y | Y | N | N |
| dassanayake, CNS drug, 2012 | Psychotropic drug overuse | Y | Car crash | Y | Y | Y | Y | N |
| De wals, JAMA, 2012 | H1N1 influnza vaccine | Y | GBS | Y | Y | Y | Y | Y |
| Donegan, Vaccine, 2013 | HPV vaccine | Y | Chronic fatigue syndrome | Y | N | Y | Y | Y |
| Douglas, BMJ, 2013 | Orlistat | unclear | Acute liver injury | Y | Y | Y | Y | Y |
| fife, Drug Saf, 2014 | Fluoroquinolones | Y | Retinal detachment | Y | Y | Y | Y | Y |
| hambidge, Pediatrics, 2012 | Trivalent influenza vaccine | Y | Hospitalisation with sickle cell crisis | Y | Y | Y | Y | Y |
| Hambidge, Vaccine, 2011 | Trivalent influenza vaccine | Y | Hospitalisation with sickle cell crisis | Y | Y | Y | Y | Y |
| Hanf, Vaccine, 2013 | MMR vaccine | Y | Febrile convulsion | Y | Y | Y | Y | Y |
| Hawken, Am J Epid, 2012 | Acellular pertussis vaccine | Y | Hospital admission and ER visits | Y | Y | Y | Y | Y |
| huang, vaccine 2014 | H1N1 influnza vaccine | Y | Spontaneous abortion | Y | Y | Y | Y | Y |
| huang, Plos One, 2013 | H1N1 influnza vaccine | Y | GBS, central demyelinisating disease, convulsion, encephalitis, stroke, idiopathic thrombocytopenia | Y | Y | Y | Y | Y |
| macartney, vaccine, 2014 | MMR and varicella vaccines | Y | Febrile seizure | Y | Y | Y | Y | Y |
| man, pediatrics, 2014 | Methylphenidate | Y | ED admission for traumatism | Y | Y | Y | Y | N |
| masclee, gastroenterol, 2014 | Combinaison of NSAID, aspirin, anticoagulant, SSRI, antihypertensive | Y | UGIB | Y | Y | Y | Y | Y |
| Mulloony, Vaccine, 2011 | Vaccines | Y | Wheezing lower respiratory diseases | Y | Y | Y | N | Y |
| Parriente, Arch Int Med, 2012 | Antidepressant initiation | Y | MI | Y | Y | Y | N | N |
| Pottegard, PDS, 2014 | Tramadol | Y | INR>4 | Y | Y | Y | N | Y |
| Pratt, Drug Saf, 2011 | Antipsychotic | Y | Hospitalisation for hip fracture or pneumonia | Y | Y | Y | Y | N |
| Pratt, Drug Saf, 2014 | Ranibizumab (inhibiteur VEGF) | Y | Ischaemic stroke | Y | Y | Y | Y | N |
| Quinn, PIDJ, 2014 | Rotavirus vaccins | Y | Intussusception | Y | Y | Y | Y | Y |
| Ramsay BMC MRM, 2013 | Proton pump inhibitors | Y | Hospitalisation for pneumonia | Y | Y | Y | Y | Y |
| Ramsay BMC MRM, 2011 | Beta blockers | Y | Hospitalisation for heart failure | Y | Y | Y | Y | N |
| Schurink, Eur J Ped 2014 | Anti HPV vaccine | Y | Migraine | Y | Y | N | Y | Y |
| Romio, Plos One 2014 | H1N1 influnza vaccine | Y | GBS | Y | Y | Y | Y | Y |
| Sun, JAMA 2012 | Combined DTaP-IPV-Hib vaccine | Y | Febrile seizures and epilepsy | Y | Y | Y | Y | Y |
| Tseng, J Int Med, 2011 | Zoster vaccine | Y | Stroke and cerebrovascular disease, cardiovascular disease, meningitis, Bell's palsy, medically attended reactions, death | Y | Y | Y | Y | N |
| Tseng, CID, 2013 | DTP vaccine | Y | Meningitis, encephalitis, cranial nerve disorders, GBS, anaphylaxis reaction | Y | Y | Y | Y | Y |
| Wilson, Vaccine, 2011 | Recommended 2mo vaccination | Y | combined end point: ED visits, hospital admissions and death | Y | Y | Y | Y | Y |
| Wilson, Vaccine, 2011 | Recommended 2mo vaccination | Y | combined end point: ED visits and hospital admissions | Y | Y | Y | Y | Y |
| Wilson,HVIT,2011 | Recommended 2mo, 4mo, 6 mo vaccination | Y | combined end point: ED visits and hospital admissions | Y | Y | Y | N | Y |
| Wilson,Vaccine, 2011 | Recommended 2mo, 4 mo, 6mo and 12 mo vaccination | Y | combined end point: ED visits and hospital admissions | Y | Y | Y | Y | Y |
| Wiljaars,BMJO, 213 | Antidepressants | Y | "Successful" suicide, suicide attempts and self-harm, suicidal ideation | Y | Y | Y | Y | Y |
| Ueyama, H. Drug Saf. 2014 | Palivizumab | Y | Hospitalisation for a known adverse reaction to pavilizumab | Y | Y | Y | Y | Y |
| Hambidge, S. Pediatrics. 2014 | Recommended vaccination in the first 2 years of life | Y | Seizure | Y | Y | Y | Y | Y |

GLP-1: glucagon-like peptide 1; MMR: measles, mumps, and rubella; ED: emergency department; CV: cardiovascular events; GBS: Guillain-Barré syndrome

# Supplementary Material S5: Validity assumptions for the use of a self-controlled design. Sensitivity analysis when unclear assumptions are considered invalid.

|  | Case-crossover | Self-controlled case-series |
| --- | --- | --- |
|  | N=49 | N=51 |
| **Major asumptions:** |  |  |
| **All major validity assumptions fulfilled^1^** | **32 (60)** | **47 (85)** |
|  |  |  |
| **Minor asumptions:** |  |  |
| Assumption of the same opportunity of exposure fulfilled^2^ | 31/32 (97) | - |
| Assumption of the absence of exposure time trend (or design adapted) fulfilled | 9/32 (28) |  |
| Assumption of independence between consecutive events when recurrent (or design adapted) fulfilled^3^ | - | 39/47 (83) |
| Assumption of the event-independent exposure (or design adapted) fulfilled^4^ | - | 35/47 (74) |
| Assumption of the absence of censoring of the observation period fulfilled^5^ | - | 39/47 (83) |
| **All minor validity assumptions for the use of a self-controlled design fulfilled (among papers with valid major assumptions)^6^** | **9/32 (28)** | **25/47 (53)** |
|  |  |  |
| **All major and minor validity assumptions for the use of a self-controlled design fulfilled (among all papers)** | **9/49 (18)** | **25/51 (49)** |
| Data are reported as N (%)  ^1^For case-crossover, those characteristics consist in a transient or intermittent exposure, an abrupt onset event, and a rare event. For self-controlled case series, they consist in a transient or intermittent exposure, an abrupt onset event, and a rare and/or recurrent event. Unclear exposures were considered as sustained.  ^2^Opportunity of exposure is the same during case and control time periods.  ^3^This assumption is fulfilled for papers studying non recurrent events, or recurrent events with independence between consecutive events, or non-independent recurrences where the design is adapted, or recurrent events for which independence is unclear.  ^4^This assumption is fulfilled for papers studying exposure whose probability is not affected by previous events, or papers using an adapted design when probability of exposure is affected by a previous event, or unclear event-independent exposures.  ^5^This assumption is fulfilled for papers studying outcomes that do not censors the observation period by affecting the short term mortality probability, or papers using an adapted design when the observation period can be censored by the outcome  ^6^For case-crossover, the opportunity for exposure should be the same during the case and control time periods, and there should not be any time trend in exposure. For self-controlled case series, two consecutive events should be independent if they are recurrent; the probability of further exposure should not be affected by a previous event; the event should not affect the short-term mortality probability neither censor the observation period. In case of violation of one of these assumptions, the design should be adapted. | | |

# Supplementary Material S6. Validity assumptions for the use of a self-controlled design. Sensitivity analysis after excluding articles for the same series.

|  | Case-crossover | Self-controlled case-series |
| --- | --- | --- |
|  | N=49 | N=51 |
| Type of studied exposure |  |  |
| Intermittent/transient | 34 (69) | 45 (88) |
| Sustained | 11 (23) | 5 (10) |
| Unclear | 4 (8) | 1 (2) |
| Event’s onset |  |  |
| Abrupt | 48 (98) | 49 (96) |
| Insidious | 1 (2) | 2 (4) |
| Rare event | 46 (94) | 44 (86) |
| Recurrent event (for self-controlled case series only) | - | 38 (75) |
| Rare and/or recurrent event (for self-controlled case series only) | - | 51 (100) |
| **All major validity assumptions fulfilled^1^** | **35 (71)** | **44 (86)** |
| Assumption of the same opportunity of exposure fulfilled^2^ | 34/35 (97) | - |
| Time trend in exposure |  |  |
| Yes  *In case of exposure time trend, the design is adapted* | 8/35 (23)  *5/8 (63)* | - |
| No | 5/35 (14) | - |
| Unclear | 22/35 (63) | - |
| Assumption of the absence of exposure time trend (or design adapted) fulfilled | 32/35 (91) |  |
| Independence between consecutive events |  |  |
| Yes | - | 5/44 (11) |
| No  *For non-independent recurrences, the design is adapted* | - | 14/44 (32)  *13/14 (93)* |
| Unclear | - | 13/44 (30) |
| Not applicable (for non-recurrent events) | - | 12/44 (27) |
| Assumption of independence between consecutive events when recurrent (or design adapted) fulfilled^3^ | - | 43/44 (98) |
| Event-independent exposure |  |  |
| Yes | - | 5/44 (11) |
| No  *Of which the design is adapted* | - | 35/44 (80)  *30/35 (86)* |
| Unclear | - | 4/44 (9) |
| Assumption of the event-independent exposure (or design adapted) fulfilled^4^ | - | 39/44 (89) |
| The event affect the short term mortality probability (censoring of the observation period) | - |  |
| Yes  *Of which the design is adapted* | - | 12/44 (27)  *4/12 (33)* |
| No | - | 32/44 (73) |
| Assumption of the absence of censoring of the observation period fulfilled^5^ | - | 36/44 (82) |
| **All minor validity assumptions for the use of a self-controlled design fulfilled (among papers with valid major assumptions)^6^** | **31/35 (89)** | **31/44 (70)** |
| **All major and minor validity assumptions for the use of a self-controlled design fulfilled (among all papers)** | **31/49 (63)** | **31/51 (61)** |
| Data are reported as N (%)  ^1^For case-crossover, those characteristics consist in a transient or intermittent exposure, an abrupt onset event, and a rare event. For self-controlled case series, they consist in a transient or intermittent exposure, an abrupt onset event, and a rare and/or recurrent event. Unclear exposures were considered as intermittent.  ^2^Opportunity of exposure is the same during case and control time periods.  ^3^This assumption is fulfilled for papers studying non recurrent events, or recurrent events with independence between consecutive events, or non-independent recurrences where the design is adapted, or recurrent events for which independence is unclear.  ^4^This assumption is fulfilled for papers studying exposure whose probability is not affected by previous events, or papers using an adapted design when probability of exposure is affected by a previous event, or unclear event-independent exposures.  ^5^This assumption is fulfilled for papers studying outcomes that do not censors the observation period by affecting the short term mortality probability, or papers using an adapted design when the observation period can be censored by the outcome  ^6^For case-crossover, the opportunity for exposure should be the same during the case and control time periods, and there should not be any time trend in exposure. For self-controlled case series, two consecutive events should be independent if they are recurrent; the probability of further exposure should not be affected by a previous event; the event should not affect the short-term mortality probability neither censor the observation period. In case of violation of one of these assumptions, the design should be adapted. | | |

1. Not reported or unclear [↑](#footnote-ref-2)
2. Population covered by the database, and from which the study sample is taken [↑](#footnote-ref-3)
3. For example in the study of rosiglitazone and the risk of cardiovascular disease, number of diabetic patients in the database, as reported in the article [↑](#footnote-ref-4)
4. In terms of demographics [↑](#footnote-ref-5)
5. Yes in case of pharmaceutical dispensation database. No in case of reimbursement database [↑](#footnote-ref-6)
6. In terms of demographics [↑](#footnote-ref-7)
7. Reported in the article as not collected in the database [↑](#footnote-ref-8)
8. Yes in case of administrative database [↑](#footnote-ref-9)
9. Yes in case of different administrative database. May be not independently if the exposure is collected by interview or questionnaire, or in the same database [↑](#footnote-ref-10)
10. For cohort studies, number of exposed patients over the total number of eligible patients ; for case-control studies, prevalence in the control group [↑](#footnote-ref-11)
11. Not applicable if several events [↑](#footnote-ref-12)
12. For example treatment of asthma crisis [↑](#footnote-ref-13)
13. For example antibiotics [↑](#footnote-ref-14)
14. The risk of exposure vary over time (for example : the medication has market a authorization recently) [↑](#footnote-ref-15)
15. The exposure is independent of the outcome if the drug can be prescribed after the event. It is not independent if the outcome is death or a contra-indication of the drug [↑](#footnote-ref-16)
16. Generally, not in this study particularly [↑](#footnote-ref-17)
17. Yes in case of event collected in a different database as the exposure, or in reimbursement database. May be not independently if the outcome is collected by interview or questionnaire or in the same database as the exposure, mainly in prescription or general practice database [↑](#footnote-ref-18)
18. Thus the study methodology can be replicated [↑](#footnote-ref-19)
19. e.g. prescription of insuline for diagnosis of diabetes [↑](#footnote-ref-20)
20. In case-control design, number of total cases included divided by the number of eligible patients in the database over the study period [↑](#footnote-ref-21)
21. i.e. can be cured [↑](#footnote-ref-22)
22. i.e. cannot be cured [↑](#footnote-ref-23)
23. With sequellae or relapses [↑](#footnote-ref-24)
24. The risk of event varies over time (for example: the incidence was increased after a given date). The risk of event varying with age should not be considered. [↑](#footnote-ref-25)
25. e.g. an other antidiabetic, or antipsychotic drug [↑](#footnote-ref-26)
26. Yes in case of population-based study. A database covering special population can’t be considered as representative. [↑](#footnote-ref-27)
27. e.g. insuline precribed for diabetic patients [↑](#footnote-ref-28)
28. No if PS used in a secondary or sensitivity analysis, or if PS used but no primary analysis can be identified [↑](#footnote-ref-29)
29. Several definitions of the exposure can be considered as sensitivity analyses, even if not specified. [↑](#footnote-ref-30)
30. Should be described in the method section [↑](#footnote-ref-31)
31. e.g. refusals, drops out, cases not matched [↑](#footnote-ref-32)
32. Either for cumulative dose, or duration of use [↑](#footnote-ref-33)
33. Exposed case are case whose prescription is given during risk period [↑](#footnote-ref-34)
34. Unexposed case are case whose prescription is not given during risk period, or case with no prescription [↑](#footnote-ref-35)
